# Discovery of tissue-specific proteomic signatures in juvenile dermatomyositis highlights pathways reflecting persistent disease activity, clinical heterogeneity, and myositis-specific autoantibody subtype

**DOI:** 10.1016/j.ard.2025.07.020

**Published:** 2025-09-06

**Authors:** Jessica Neely, Sara E. Sabbagh, Jeffrey Dvergsten, Chioma Madubata, Celine C. Berthier, Zilan Zheng, Christine Goudsmit, Sophia Matossian, Sean P. Ferris, Gabriela K. Fragiadakis, Marina Sirota, J. Michelle Kahlenberg, Hanna Kim, Jessica L. Turnier

**Affiliations:** 1 Department of Pediatrics, Division of Rheumatology, University of California, San Francisco, San Francisco, CA, USA; 2 Department of Pediatrics, Division of Rheumatology, The Medical College of Wisconsin, Milwaukee, WI, USA; 3 Department of Pediatrics, Division of Rheumatology, Duke University, Durham, NC, USA; 4 Department of Internal Medicine, Division of Nephrology, University of Michigan, Ann Arbor, MI, USA; 5 Department of Pediatrics, Division of Rheumatology, University of Michigan, Ann Arbor, MI, USA; 6 Department of Pathology, University of Michigan, Ann Arbor, MI, USA; 7 Department of Medicine, Division of Rheumatology, University of California, San Francisco, San Francisco, CA, USA; 8 Bakar Computational Health Science Institute, University of California, San Francisco, San Francisco, CA, USA; 9 Department of Internal Medicine, Division of Rheumatology, University of Michigan, Ann Arbor, MI, USA; 10 Juvenile Myositis Pathogenesis and Therapeutics Unit, National Institute of Arthritis Musculoskeletal and Skin Diseases (NIAMS), National Institutes of Health (NIH), Bethesda, MD, USA

## Abstract

**Objectives:**

Juvenile dermatomyositis (JDM) is a heterogeneous autoimmune condition needing targeted treatment approaches and improved understanding of molecular mechanisms driving clinical phenotypes. We utilised exploratory proteomics from a longitudinal North American cohort of patients with new-onset JDM to identify biological pathways at disease onset and follow-up, tissue-specific disease activity, and myositis-specific autoantibody (MSA) status.

**Methods:**

We measured 3072 plasma proteins (Olink panel) in 56 patients with JDM within 12 weeks of starting treatment (from the Childhood Arthritis and Rheumatology Research Alliance Registry and 3 additional sites) and 8 paediatric controls. Twenty-four patients with JDM who had 6-month follow-up samples were assessed. We identified differentially expressed proteins (DEPs) between groups by fitting linear mixed effects models and associated DEPs with validated disease activity measures. We assessed for cell/tissue specificity using the Human Protein Atlas and JDM muscle single nuclei and skin single-cell transcriptomic datasets. Differences within MSA subgroups were also analysed.

**Results:**

We uncovered persistent dysregulation of innate immune activation, cell death, and redox signalling at 6 months despite multidrug immunosuppression. By leveraging tissue and cell-specific proteomes, we identified overrepresentation of circulating endothelial proteins associated with disease activity and verified endothelial cell marker expression in JDM muscle and skin. We discovered pathways associated with MSA subtypes that reflect JDM phenotypes. NXP2+ JDM-associated proteins reflected angiogenesis and extracellular matrix remodelling and were expressed in endothelial cells and fibroblasts. MDA5+ JDM was associated with circulating type III interferon and surfactant proteins.

**Conclusions:**

These proteomic findings will inform future biomarker and treatment development considering the unique tissue- and autoantibody-associated inflammation in JDM.

## INTRODUCTION

Juvenile dermatomyositis (JDM) is a heterogeneous immunemediated disease characterised by muscle weakness, rash, and vasculopathy. Children with JDM often have refractory disease, with patients experiencing chronic muscle weakness, persistent rashes, and long-term disability [1]. Reliable, individual, and tissue-specific biomarkers to monitor disease activity are lacking. Currently, blood biomarkers used to assess clinical disease activity, such as creatinine kinase (CK) and lactate dehydrogenase, reflect only muscle involvement and are often unreliable [2,3]. Other clinically available biomarkers, including von Willebrand factor antigen and neopterin, have variable prevalence and myositis-specific autoantibody (MSA) association [4–7]. While many studies have demonstrated a high interferon (IFN) signature in JDM [8–12] associated with increased disease activity [8,10,11,13], these IFN markers may not accurately reflect ongoing inflammation in patients with chronic/treatmentrefractory disease or low disease activity [9,14]. A subset of patients also has an incomplete response to IFN-pathway targeting [15]. To improve outcomes for children with JDM, identification of biomarkers reflective of biological pathways that encompass the complexity of JDM multiorgan pathophysiology and also a personalised approach to disease management linking disease pathophysiology to phenotype is imperative.

The peripheral blood transcriptome does not generally reflect tissue-specific information [16]; thus, several groups have utilised proteomics to investigate candidate disease activity markers [17–19]. These studies have corroborated serum biomarkers associated with systemic inflammation, such as CXCL10 and CXCL11 [17,20–22], identified galectin-9, CXCL10, and TNFR11 as predictors of poor treatment response [21,22], and found anti-MDA5 specific upregulation of type I IFN and proteasome pathways [18]. Investigations of endothelial-specific proteins have also identified VCAM-1, ANGPT2, and sVEGFR-1 (FLT-1) as disease activity biomarkers [21,22]. Recently, proteomic analysis utilising a larger, multiplexed immunoassay of 282 proteins in JDM serum detected high expression of MMP3 and GDF15 and identified IL1RL1 as a candidate global disease activity biomarker [19]. Another study applying a combined transcriptomic and proteomic analysis identified a prominent neutrophil degranulation signature in JDM [23].

Although these studies provide important clues to JDM pathophysiology, they were often constrained by low sample size, geographic homogeneity, low disease activity state with concurrent immunosuppression, or analysis of a limited number of proteins. Furthermore, contextualising the cell- and tissue-specific origin of these biomarkers and pathways has not been possible. To overcome prior limitations, we utilised an exploratory proteomics approach to assess 3072 plasma proteins in a multicentre North American cohort of 56 newly diagnosed patients with JDM and 24 paired patient samples at 6-month follow-up. We uncovered pathways not targeted by existing treatments and discovered disease activity-associated proteins that exhibit tissue- and cell-specificity in JDM muscle and skin. We revealed unique proteins underlying clinical heterogeneity by MSA phenotypes. Together, these results identify persistent dysregulation of innate immune, cell death, and redox pathways, provide a window into endothelial cell dysfunction in JDM, and highlight pathway differences by MSA.

## METHODS

### Patients

Subjects diagnosed with JDM by a paediatric rheumatologist (n = 56) and paediatric controls (CTL, n = 8) were recruited from the University of Michigan (U-M) (JDM n = 7, CTL n = 4), Duke University (JDM n = 9), the University of California San Francisco (JDM n = 12, CTL n = 4), and the Childhood Arthritis and Rheumatology Research Alliance (CARRA) Registry and Biobank (JDM n = 28). The CARRA Registry is a North American rheumatologic disease registry that enrols patients from 72 sites in the United States and Canada with JDM diagnosed within 6 months and no more than 12 weeks of treatment. No patients met criteria for another autoimmune disease. Controls were recruited from paediatric and rheumatology clinics who had no evidence of autoimmune disease, and samples were processed the same as patients with JDM at the respective sites. Institutional Review Board (IRB) approval was granted from each site and the CARRA Registry and Biobank. All subjects provided informed consent and assent as appropriate. Of 56, 24 patients had follow-up samples 6 months into treatment (CARRA n = 20 and Duke n = 4). At each visit, International Myositis Assessment and Clinical Studies Group core consensus data set components [24] were collected, including the Physician’s Global Activity Assessment (PGA), an overall assessment of disease activity on a 0 to 10 visual analogue scale (VAS).

### Proteomic analysis

We utilised the multiplexed Olink Explore 3072 Proximity Extension Assay platform to measure proteins in plasma samples (www.olink.com). The OlinkAnalyze R Package (v3.7.0) was used for downstream analyses. Protein expression in Olink data is provided as normalised protein expression (NPX) values, Olink’s arbitrary unit, on a log2 scale. After removing proteins that were designated with an assay warning by Olink (did not pass Olink’s quality control n = 175), we analysed a total of 2897 proteins (2879 unique). A principal component analysis (PCA) plot revealed a potential for site-specific effects necessitating the use of linear mixed effects (lme) models to account for site (Supplementary Fig S1). In each model, proteins with duplicate measures within the Olink Explore panel were included, and downstream results were then either averaged or filtered for the greatest estimate. To identify differentially expressed proteins (DEPs), lme models were fit to each protein using the ‘olink_lmer’ function with ‘site’ as a random effect and ‘individual’ as a random effect in models when longitudinal timepoints were included. In each model, Benjamini-Hochberg adjusted *P* values were reported and those meeting a threshold of *P*-adj < .05 were included in a posthoc analysis using the ‘olink_lmer_posthoc’ function to quantify the direction and magnitude of the protein association accounting for the random effects. Significance threshold was a post-hoc *P*-adj of <.05. A lme model analysis was used for baseline (BL) vs CTL samples, follow-up (FU) longitudinal vs CTL samples, and association of PGA VAS in all JDM samples. A paired *t*-test with ‘olink_ttest’ function was used for longitudinal samples (n = 24) (*P*-adj < .05).

### Tissue-specific analyses

The Human Protein Atlas (HPA) tissue-specific proteome tool was utilised to identify protein lists that were ‘tissue-enriched’ and ‘tissue-enhanced’ for muscle and skin [25]. The HPA cell-specific proteome tool was used to identify ‘cell type enriched’ and ‘cell type enhanced’ endothelial cell proteins. To identify tissue- and cell-associated proteins, we evaluated the intersection of these lists with disease activity-associated proteins. To determine tissue-specific markers of organ-associated clinical assessments, the intersection of the HPA tissue lists and the Olink data set (n = 243 proteins) was used to fit lme models to Childhood Health Assessment Questionnaire (CHAQ), physician muscle VAS (0–10), and physician skin VAS (0–10), with both site and individual included as random effects as above.

### MSA analysis

All MSA testing was performed using the ‘comprehensive myositis autoantibody panel’ at Oklahoma Medical Research Foundation on a clinical or research basis (omrf.org/researchfaculty/core-facilities/myositis-testing/). MSA-associated proteins were identified by fitting a lme to each protein in BL (n = 55, 1 sample overlapping between 2 sites but obtained at different time points was excluded) samples for anti-TIF1*γ*, anti-NXP2, and anti-MDA5. Only BL samples were assessed to minimise confounding by medication. One patient tested positive for both anti-NXP2 and anti-TIF1*γ* on 2 occasions and was included in both models. To increase discovery of MSA-associated proteins in a smaller number of patients, proteins reaching a *P*-adj < .1 were included in a post-hoc analysis. Those with post-hoc *P*-adj < .05 were deemed significant.

### Pathway enrichment analyses, heatmap and dot plot generation

MetaCore (www.clarivate.com) software, V24.3.71800, was used to identify the ‘process networks’ or ‘Gene Ontology Biological Process’ (GOBP) enriched in each set of DEP. Pathways with false discovery rate (FDR)<0.05 were considered significant. Heatmaps were generated using the Morpheus visualisation software from the Broad Institute (https://software.broadinstitute.org/morpheus), using the ‘transform values: subtract row mean, divide by row standard deviation’ option (which generates colour intensity based on the Row *Z*-score).

### Single-cell data

Single nuclei RNAseq data were generated from JDM (n = 6) and histopathologically normal CTL (n = 7) frozen muscle multiplexed in 2 batches using the 10 × Genomics multiome kit. A technical replicate from 1 patient was included in both batches. Freemuxlet was used to genetically demultiplex samples (https://github.com/statgen/popscle). DoubletFinder was used to remove heterotypic doublets [26]. Low-quality cells were filtered. SoupX was used for ambient RNA filtering [27]. Libraries were merged, scaled, and integrated using Harmony [28] and variable features. Single-cell RNAseq data using archival formalin-fixed, paraffin-embedded skin blocks from lesional JDM biopsies (n = 12) and CTL skin (n = 4) were generated using the 10 × Genomics Fixed RNA Profiling kit with ‘Chromium Human Transcriptome Probe Set v1.0.1’. Low-quality cells were removed. Doublet removal was performed using DoubletFinder [26], and SoupX was used for ambient RNA filtering [27]. For both datasets, Seurat (v4.3) was used to create uniform manifold approximation and projection (UMAP) embeddings and clusters were identified with the Leiden algorithm and manual annotation. Both datasets were subset to include only tissue-specific proteins of interest to evaluate cell-type-specific gene expression. N = 3 skin biopsies and n = 3 muscle biopsies were from patients in the proteomics cohort; however, sampling was not obtained at the same time.

### Patient and public involvement

Patients with JDM and their families were involved in the discussion of research priorities that led to this study. This project proposal was reviewed by a parent of a child with rheumatic disease within CARRA. Interim results were presented to patients with JDM and their families at a Cure Juvenile Myositis Town Hall.

## RESULTS

### Clinical cohort characteristics

**Figure 1.**
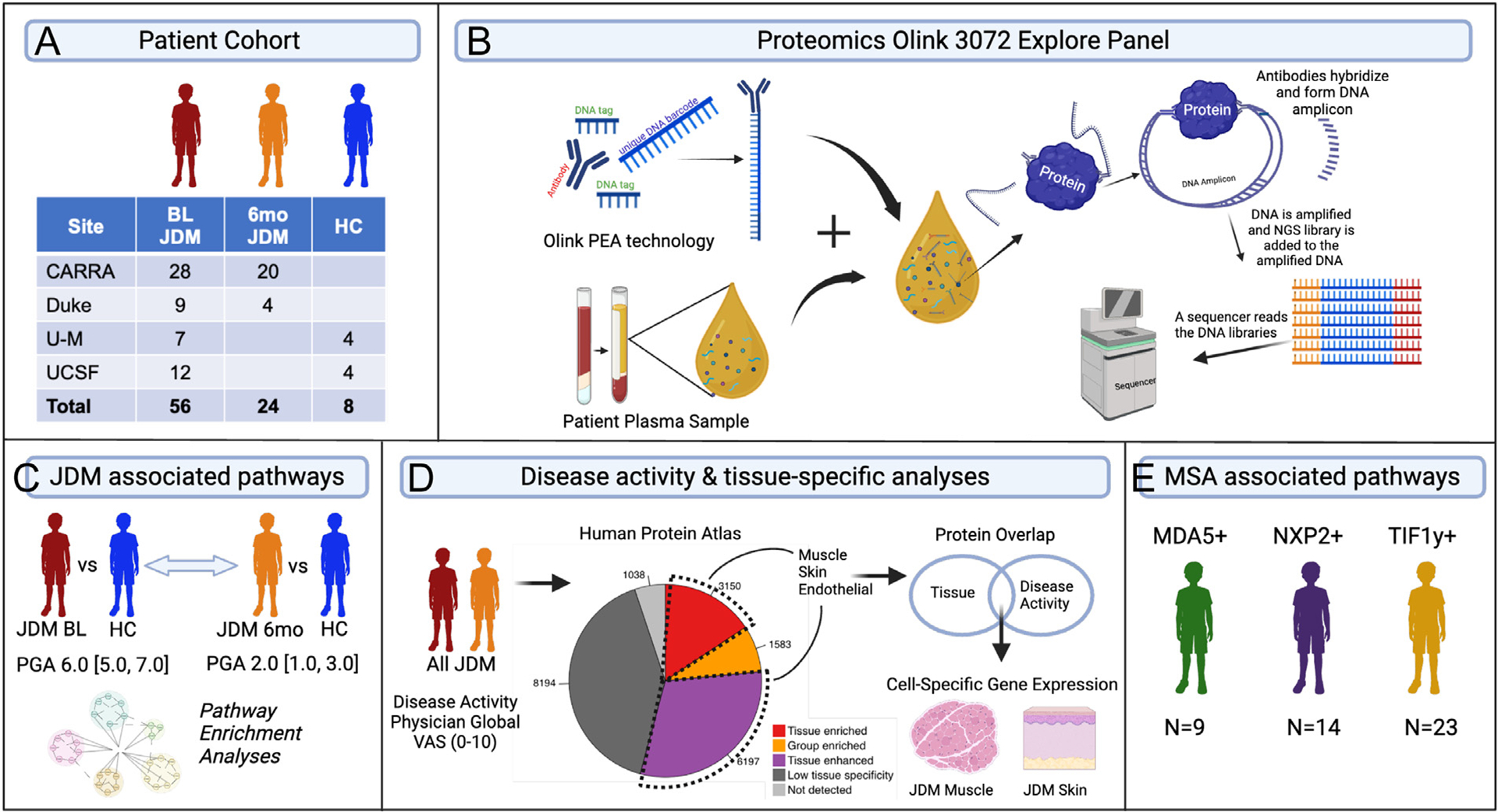
Study design overview. (A) Patient cohort details describing the number of patients with juvenile dermatomyositis (JDM) with baseline (BL) and 6-mo follow-up (6 mo) plasma samples and number of paediatric controls (CTL). (B) The Olink proximity extension assay (PEA) Explore 3072 Panel was utilised for simultaneous measurement of 3072 proteins in JDM and CTL plasma samples. Olink PEA technology harnesses antibodies labelled to DNA oligonucleotides followed by hybridisation, extension, and quantitative PCR amplification for protein quantification. Linear mixed effects model analysis identified: (C) proteins and biological pathways enriched in JDM BL or JDM 6-mo groups as compared to CTL. It also lists the physician global activity (median [IQR]), which is an overall disease activity measure on a 0 to 10 visual analogue scale (VAS). (D) Proteins associated with the global activity (PGA) in all patients with JDM at both time points. Proteins were also evaluated for either muscle, skin, or endothelial-enrichment/enhancement based on the Human Protein Atlas and evaluated for cell-type specificity in JDM muscle and skin single-nuclei and single-cell transcriptomic datasets. (E) Proteins were associated with myositis-specific autoantibody (MSA) subtype (MDA5, NXP2, TIF1γ) in BL patients with JDM. CARRA, Childhood Arthritis and Rheumatology Research Alliance; HC, healthy control; MDA5, melanoma differentiation-associated protein 5; NGS, next-generation sequencing; NXP2, nuclear matrix protein 2; PCR, polymerase chain reaction; TIF1-*γ*, transcription intermediary factor 1-gamma; UCSF, University of California San Francisco; U-M, University of Michigan.

An overview of the study design is represented in Figure 1. The patient cohort included 56 patients with JDM from a broad geographic area (Fig 1A). Patients with JDM had demographic and clinical characteristics (Table 1) similar to that reported in other registries [29,30] and moderate disease activity (median BL PGA = 6.0, IQR 5.0–7.0) (Table 2). At BL visit, 86% of patients with JDM were treatment naïve, and all had received <6 weeks of immune suppression. All but 1 patient had improvement in PGA by 6 months (Table 2). The CTL group was 50% female sex, with a median age of 7.5 years, not significantly different from the JDM group (Table 1).

### JDM proteome at disease onset and posttreatment demonstrates IFN and innate immune activation signatures

**Figure 2.**
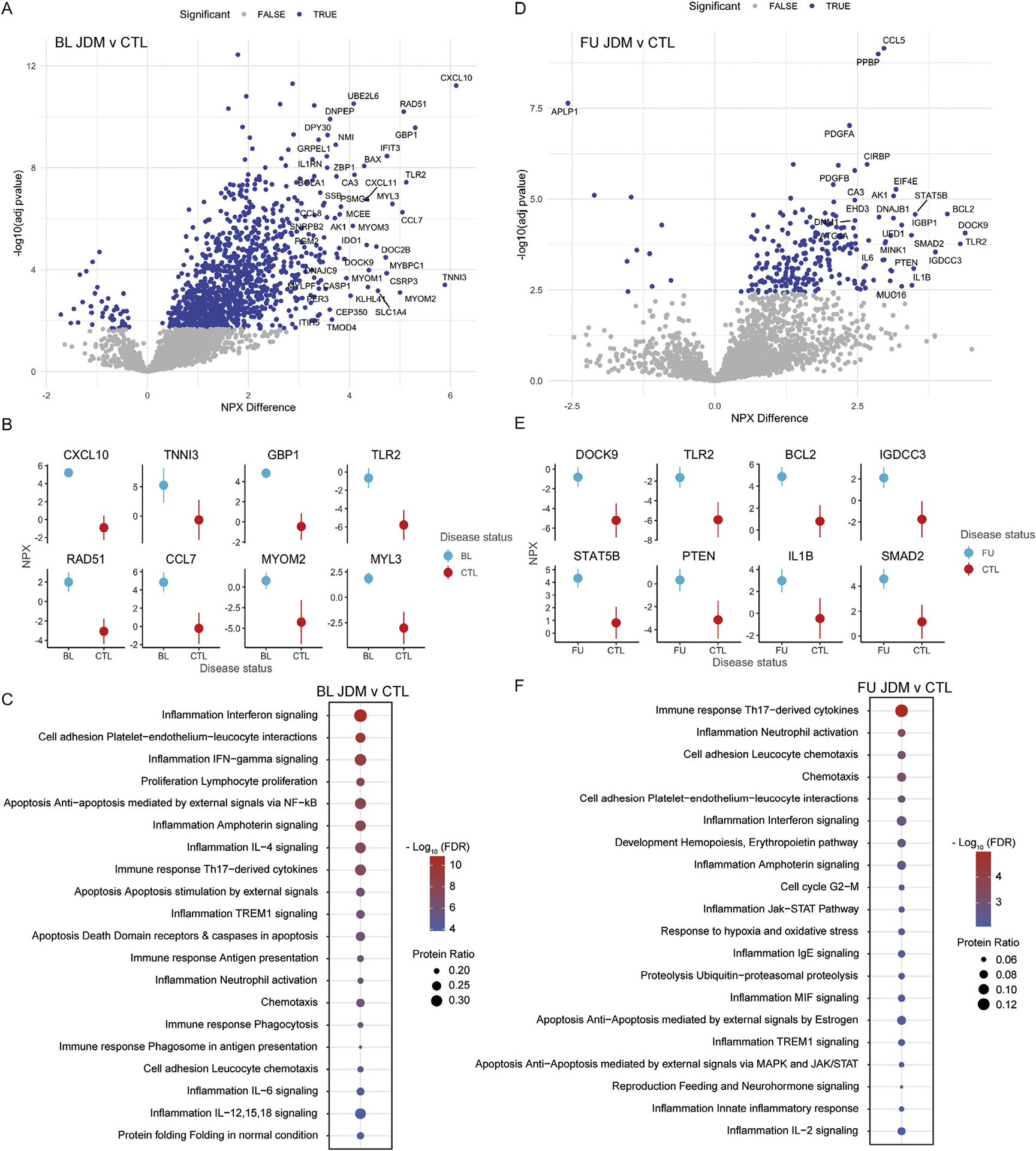
(A) Volcano plot displaying differentially expressed proteins (DEP) in baseline (BL) juvenile dermatomyositis (JDM) compared to control (CTL) using a linear mixed effects model with site as a covariate. Significance was defined as *P*-adj < .05 in the linear mixed effects model and *P*-adj < .05 in the post-hoc test. (B) Point range plots of the top 8 proteins with the greatest normalised protein expression (NPX) difference. The dots represent the fixed effect NPX estimate and the line the 95% CI summarising the NPX difference between BL JDM and CTL accounting for site effect. (C) Dot plot representing the top 20 Metacore process networks enriched at BL JDM vs CTL DEP, where dot size represents the protein ratio of DEP within each pathway and the colour of the dot represents significance defined by the FDR (false discovery rate). (D-F) Same as A-C, except for follow-up (FU) compared to CTL analyses. IFN, interferon; IL, interleukin; NF-kB, nuclear factor kappa B.

To identify biological pathways associated with disease onset, we applied a linear mixed effects model comparing BL JDM and CTL, which identified 1186 proteins (1172 unique) DEPs, including 1126 increased and 46 decreased proteins (Fig 2A, Supplementary Table S1). The top DEPs by greatest NPX difference in BL JDM were CXCL10, TNNI3, GBP1, TLR2, RAD51, CCL7, MYOM2, and MYL3 (Fig 2B). BL DEP were enriched in several inflammatory pathways and networks bridging innate and adaptive immunity, including IFN signalling, cell adhesion and chemotaxis, neutrophil activation, interleukin and Th17-derived cytokine signalling, and cell death signalling (Fig 2C, Supplementary Table S1). The same model was then used to compare 6-month FU JDM to CTL, which identified 232 DEP, including 222 increased and 10 decreased proteins (Fig 2D, Supplementary Table S2). The top DEPs at 6-month FU included DOCK9, TLR2, BCL2, IGDCC3, STAT5B, PTEN, IL1B and SMAD2 (Fig 2E). Pathway and network analysis of the DEP at 6-month FU highlighted multiple shared pathways at both BL and FU, including persistently activated inflammatory pathways such as Th17-derived cytokine and IFN signalling, neutrophil activation and chemotaxis, as well as oxidative stress, cell death, and ubiquitin-proteasome pathway activation (Fig 2C, Supplementary Table S2). Due to few number of CTL samples (n = 8) in these analyses, PCA plots comparing site and disease effects with all proteins to only those significant in the analyses were compared visually and by analysis of variance of PC1 and PC2, which suggested DEPs were driven more by biological rather than site-specific effects (Supplementary Figs S2 and S3).

### Posttreatment JDM proteome highlights persistent dysregulation of innate immune, cell death, and redox signalling

**Figure 3.**
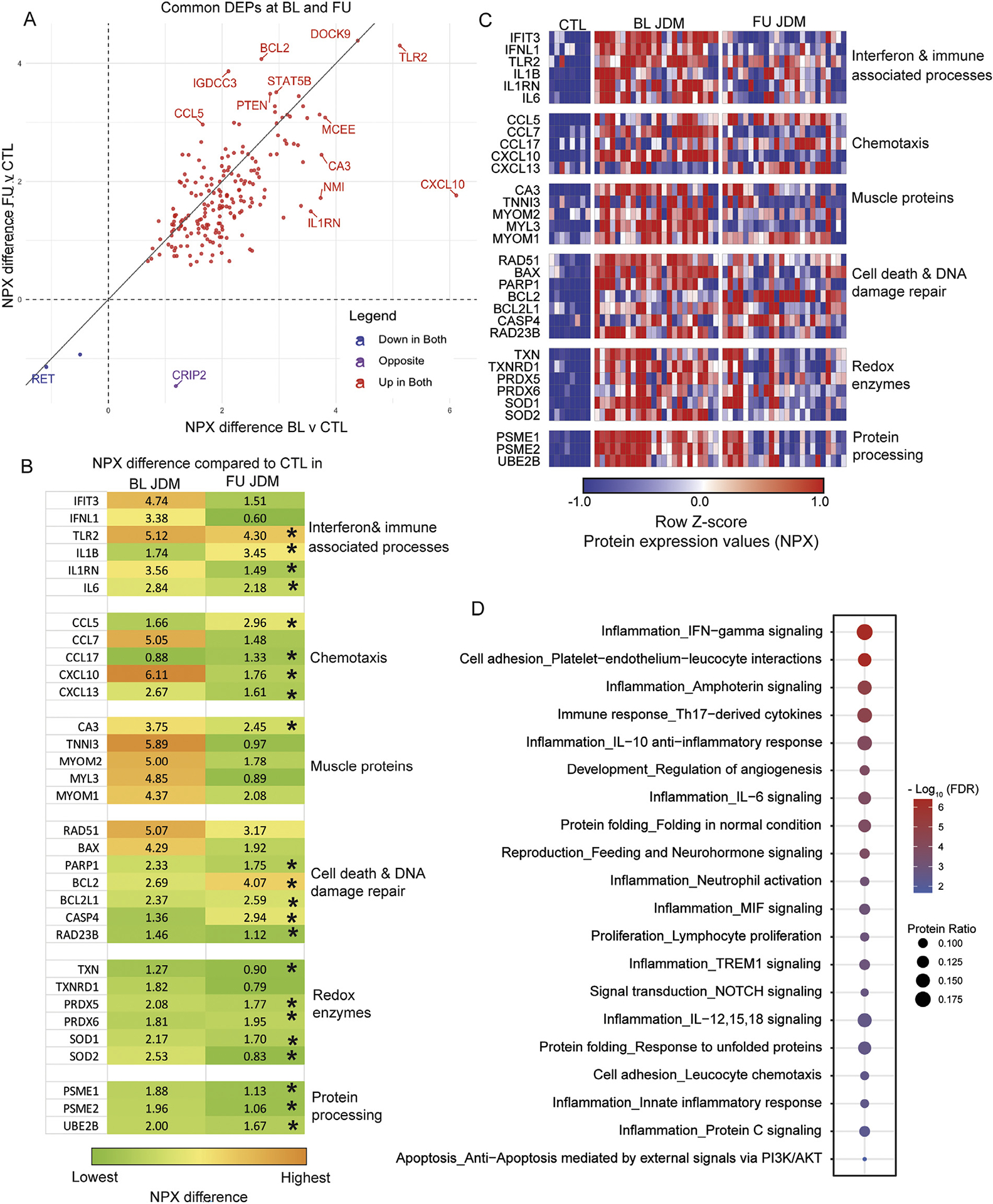
(A) Scatter plot comparing NPX difference of 196 common DEPs in BL compared to CTL (x-axis) and FU compared to CTL (y-axis). (B) Heatmap displaying the NPX difference between BL JDM and CTL and FU JDM and CTL for select DEPs at BL JDM of interest grouped by biological processes to highlight change in these proteins from BL to FU. Asterisks are used to indicate significance for the FU compared to CTL analysis. Of note, IL1B is not differentially expressed in BL JDM but was included to highlight the increase in NPX difference at 6 months. (C) Heatmap displaying NPX *z*-score expression of select proteins in 24 paired samples (and 8 CTL samples) grouped by biological pathways of interest. (D) Dot plot representing Metacore process networks enriched in the set of nonsignificant proteins in a paired analysis of n = 24 samples with BL and FU measurements, where dot size represents the protein ratio of DEP within each pathway and the colour of the dot represents significance as defined by the FDR (false discovery rate). BL, baseline; CTL, control; DEP, differentially expressed protein; FU, follow-up; IFN, interferon; IL, interleukin; JDM, juvenile dermatomyositis; NPX, normalised protein expression.

To identify proteins persistently dysregulated at 6 months posttreatment, we compared the intersection of DEP from BL vs CTL and from FU vs CTL, which revealed that 83% (976/1172) of BL DEP were no longer differentially expressed at FU. However, 84% (196/232) of the 6-month FU DEP were also differentially expressed at BL (Fig 3A), highlighting persistent dysregulation of some DEP. Whereas many proteins related to muscle structure (MYOM2, MYL3) and IFN signalling (IFIT3, IFNL1) normalised at 6 months, those related to innate immunity (TLR2, IL1RN, IL6), oxidation-reduction (redox) enzymes (PRDX5/6, SOD1/2), protein processing (PSME1/2, UBE2B) and DNA damage repair (RAD23B, PARP1), remained differentially expressed (Fig 3B). There was also a shift in the expression ratio of specific proteins at FU with opposing roles within biological pathways. For instance, in the IL1 signalling pathway, IL1B was newly differentially expressed at FU, with IL1RN (IL1 receptor antagonist) decreasing (Fig 3B). There were similar shifts in cell death signalling proteins: at 6-month FU, the proapoptotic protein, BAX, was no longer increased, whereas the anti-apoptotic protein BCL2 further increased [31] (Fig 3B). We also noted an inverse shift in chemotactic proteins (CCL5 further increasing whereas CCL7 was decreasing) (Fig 3B). The increase in IL1B at 6 months was intriguing, so we further evaluated IL1B expression in the public peripheral blood mononuclear cell (PBMC) single-cell RNAseq dataset to leverage the longitudinal nature of this dataset finding that, in fact, IL1B expression, which is restricted to CD14+ monocytes, is increased in inactive patients with JDM compared to treatment-naïve JDM (Supplementary Fig S4). To determine if patients treated more aggressively might have different profiles, we performed a sensitivity analysis removing 5 patients who received either mycophenolate or cyclophosphamide, which did not significantly change the results (Supplementary Fig S5).

### JDM paired proteome analysis at disease onset and posttreatment reveals persistent inflammation and angiogenesis

To determine if similar DEP changes occurred within individuals, we next used the 1186 DEP (1172 unique) identified in BL JDM vs CTL in a paired analysis with BL vs 6-month FU (n = 24) (Supplementary Table S3), which revealed similar shifts in dysregulated pathways at an individual level. Fifty-eight per cent (691/1186) of DEP from the prior BL JDM vs CTL analysis showed differential expression at 6-month FU within individuals, with nearly all showing decreased expression (97%, 669/691). Notably, 42% (499/1186) of proteins had no significant change at 6-month FU in individuals, despite ongoing treatment. Enrichment analysis of the 496 unique, nonsignificant or unchanged proteins within individuals between BL and FU highlighted many of the same pathways identified in the prior analysis, such as Th17-derived cytokine signalling, neutrophil activation, protein folding and processing, cell death, endothelial-immune cell interactions, and regulation of angiogenesis (Fig 3C,D), supporting the findings using the entire dataset.

### Disease activity-associated proteins reflect IFN signalling, muscle inflammation, cell death, and endothelial-immune cell interaction

**Figure 4.**
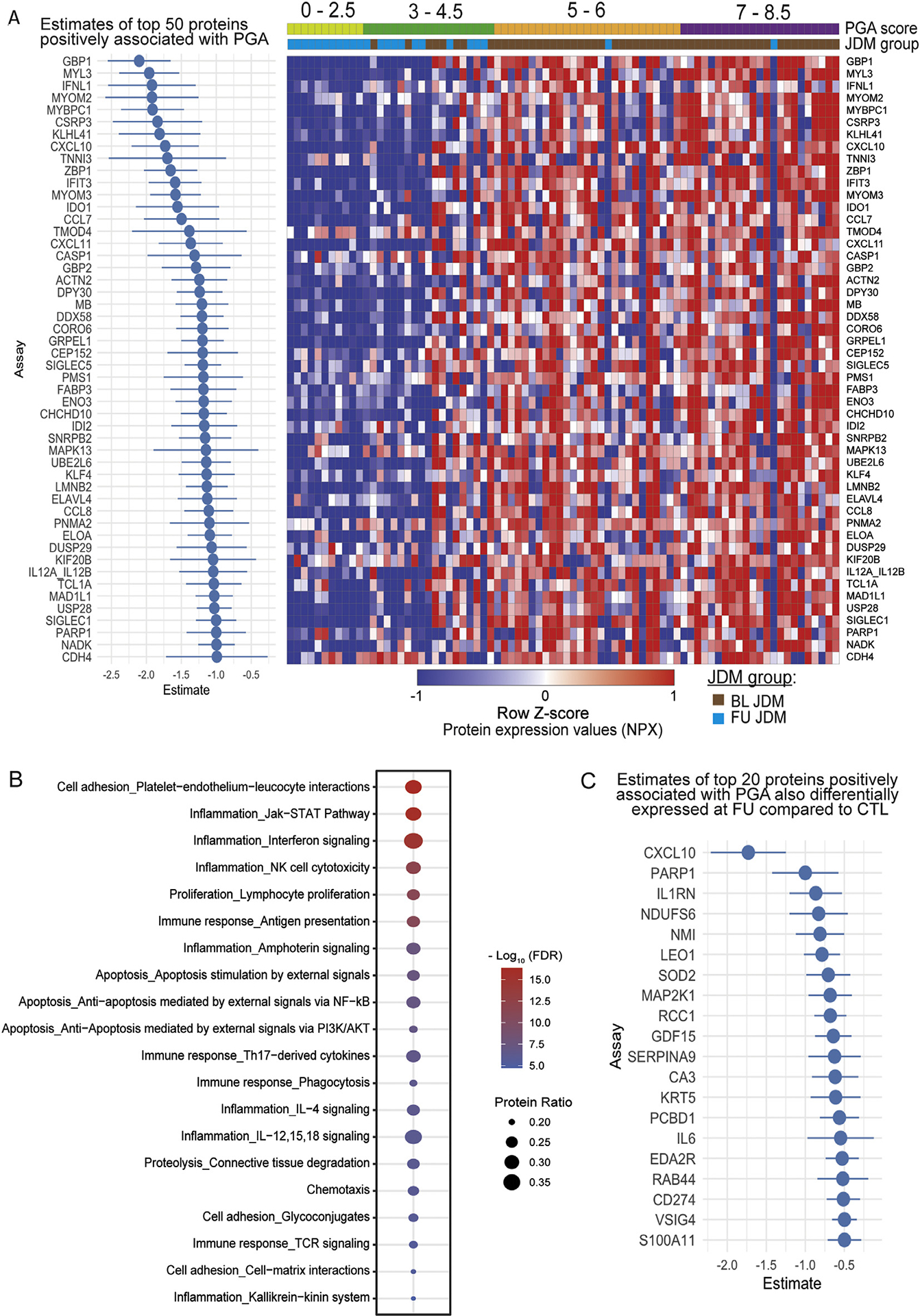
(A) (Left) Point range plot of the top 50 proteins with the greatest estimates in a linear mixed effects model with post-hoc testing evaluating the association with disease activity measured by the physician global assessment (PGA) and (right) a heatmap of individual-level z-score NPX expression of this set of proteins. The dot represents the fixed effect NPX estimate, and the line the 95% CI summarising the association with PGA, accounting for site effect and individual, where negative estimates represent proteins positively associated with PGA. A PGA score of 8.5 was the maximum value in the cohort. (B) Dot plot representing Metacore process networks enriched in PGA-associated proteins where dot size represents the percentage of DEPs within that pathway and colour represents significance defined by the FDR (false discovery rate). (C) Point range plot displaying the top 20 proteins with the greatest estimates from the PGA analysis that are also differentially expressed in the FU compared to CTL analysis. BL, baseline; CTL, control; DEP, differentially expressed protein; IL, interleukin; FU, follow-up; JDM, juvenile dermatomyositis; NF-kB, nuclear factor kappa B; NK, natural killer; NPX, normalised protein expression; TCR, T cell receptor.

Next, we fit a model to identify proteins associated with global disease activity by PGA of 80 JDM samples, including paired measurements of the same individual. We identified 1075 proteins (1062 unique) associated with PGA (Fig 4A, Supplementary Table S4). Many top proteins associated with PGA reflected IFN signalling (GBP1, IFNL1, CXCL10) and muscle structure and function (MYOM2, MYL3, MYBPC1) (Fig 4A). Top enriched pathways included endothelial cell interactions, IFN signalling, NK cytotoxicity, Th17-derived cytokine signalling, apoptosis, chemotaxis, and proteolysis (Fig 4B). Because the initial analysis revealed that many DEP normalise by 6 months, we investigated potential markers of lower disease activity. To do this, we identified the intersection of proteins associated with disease activity also differentially expressed at 6-month FU compared to CTL, identifying CXCL10, PARP1, IL1RN, SOD2, and IL6 as candidate markers that can potentially detect not only higher baseline disease activity but also lower persistent disease activity (Fig 4C).

### Candidate markers of tissue-specific disease activity reflect endothelial cell dysfunction

**Figure 5.**
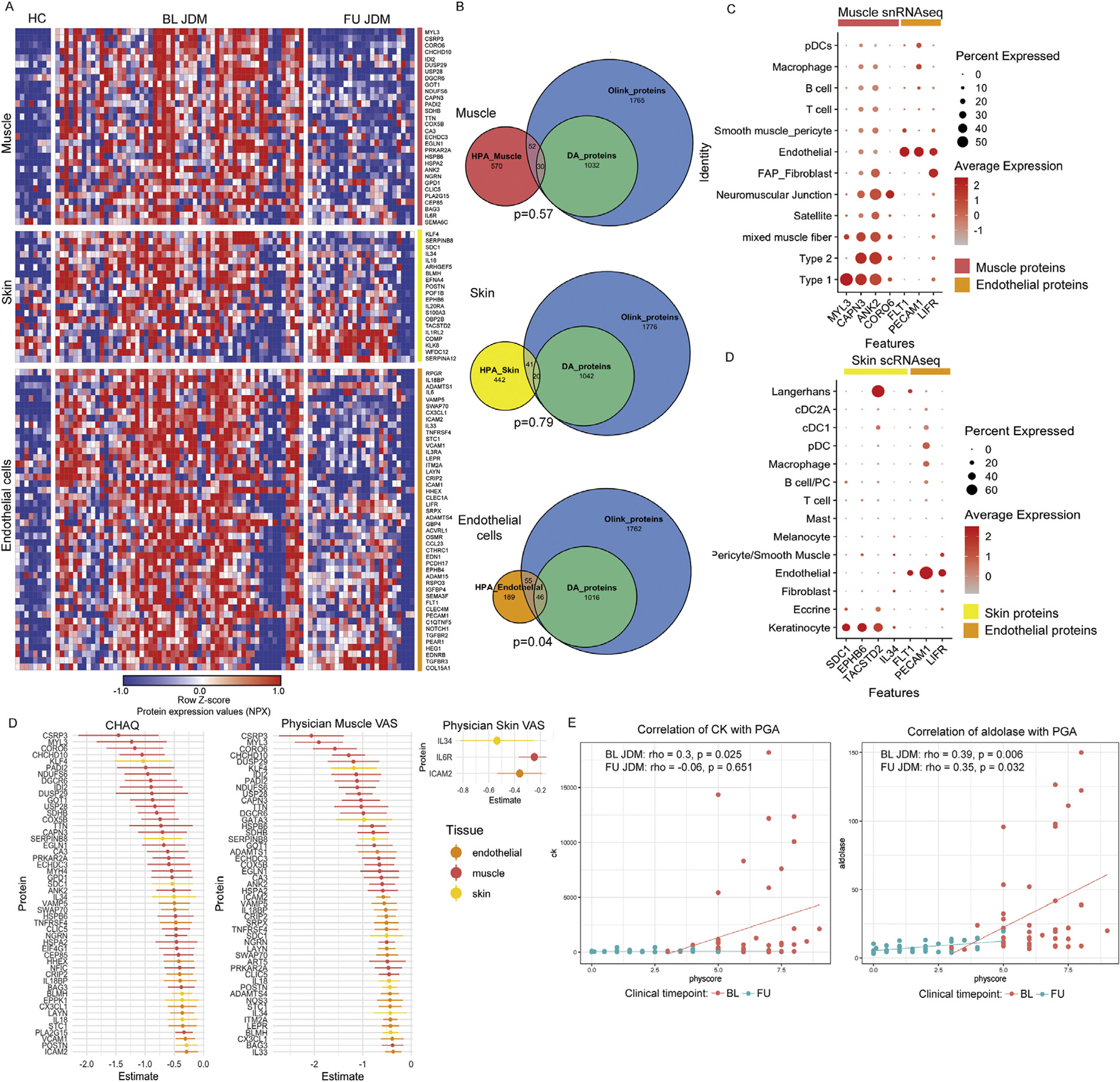
(A) Heatmap displaying individual-level *Z*-score normalised protein expression (NPX) expression of disease activity-associated proteins overlapping with tissue-specific proteome lists from the Human Protein Atlas (HPA) organised by tissue and ordered by decreasing estimates within each tissue. (B) Euler diagrams showing the overlap between tissue-specific lists from HPA, disease activity (DA) associated proteins (n = 1075) and the complete Olink protein dataset (n = 2897). *P* values represent the significance of a hypergeometric test to assess for enrichment of the tissue-specific lists within the intersection of these lists. (C, D) Dotplots displaying average gene expression (indicated by colour) and per cent of cells (indicated by size of dot) expression for select markers in (C) single nuclei RNA sequencing data from muscle (n = 6 JDM, n = 7 control, 26,638 total nuclei) and (D) single-cell RNA sequencing data from skin (n = 12 JDM, n = 4 control, 91,216 total cells). (D) Point range plots displaying the top proteins associated with Child Health Assessment Questionnaire (CHAQ), physician muscle visual analogue score (VAS), or physician skin VAS from linear mixed effects models associating with tissue-specific markers as defined by HPA represented in the Olink dataset where the dot represents the fixed effect NPX estimate and the line represents the 95% CI summarising the association with each outcome. Proteins are coloured by the associated tissue. (E) Scatter plots demonstrating the Spearman correlation (rho) between creatinine kinase (CK) (left) and aldolase (right) and the PGA score for BL samples and FU samples. BL, baseline; HC, healthy control; FU, follow-up; JDM, juvenile dermatomyositis; PGA, physician global assessment.

To identify putative tissue-specific or cell-specific disease activity markers, we found the intersection of PGA-associated proteins with the HPA [25] ‘tissue enriched’ and ‘tissue enhanced’ protein lists for muscle, skin, and endothelial cells (Fig 5A,B, full list of HPA proteins included in Supplementary Table S5). We identified 30 muscle, 20 skin, and 46 endothelial cell-specific disease activity-associated proteins (Fig 5A,B, Supplementary Table S5). A hypergeometric test was performed for each tissue, which revealed that only endothelial cell-specific proteins were over-represented among disease activity-associated proteins (*P* = .04, uncorrected) (Fig 5B).

To validate protein specificity within JDM target tissues and investigate gene expression at the single-cell level, we utilised a single nuclei RNAseq muscle dataset (n = 6 JDM, n = 7 CTL, 26,638 total nuclei) and a single-cell RNAseq skin dataset (n = 12 JDM, n = 4 CTL, 91,216 cells) generated by our group. Several disease activity-associated proteins exhibited tissue- and cell-specific gene expression (Fig 5C,D). *MYL3* was specifically expressed in type 1 muscle fibres, whereas *CAPN3* and *ANK2* were more broadly expressed in multiple muscle fibre types (Fig 5C). Skin-related markers were primarily expressed in keratinocytes (Fig 5D). Interestingly, endothelial-related genes, including *FLT1*, also known as vascular endothelial growth factor receptor 1 (*VEGFR1*), were expressed in both muscle and skin endothelial cells (Fig 5C,D).

To determine if tissue-specific proteins associated with clinical muscle and skin measures, we fit models using the tissue- or cell-specific proteins from HPA also represented in the Olink panel (n = 243 proteins, Supplementary Table S6). While muscle-specific proteins are most strongly associated with muscle disease measures (CHAQ, muscle VAS) (Fig 5D), a similar number of endothelial-specific proteins significantly associated with these outcomes (Supplementary Table S6). Only 3 proteins were significantly associated with physician skin VAS, including IL34, a skin-specific marker, as the top associated protein (Fig 5D). CK and aldolase, clinical muscle enzymes, demonstrated low Spearman correlation with PGA, with CK being particularly poor at FU (Fig 5E), highlighting the need for improved markers of tissue inflammation, particularly with lower disease activity.

### Anti-NXP2 JDM is associated with endothelial and fibroblast-associated proteins and angiogenesis and extra-cellular matrix remodelling pathways

**Figure 6.**
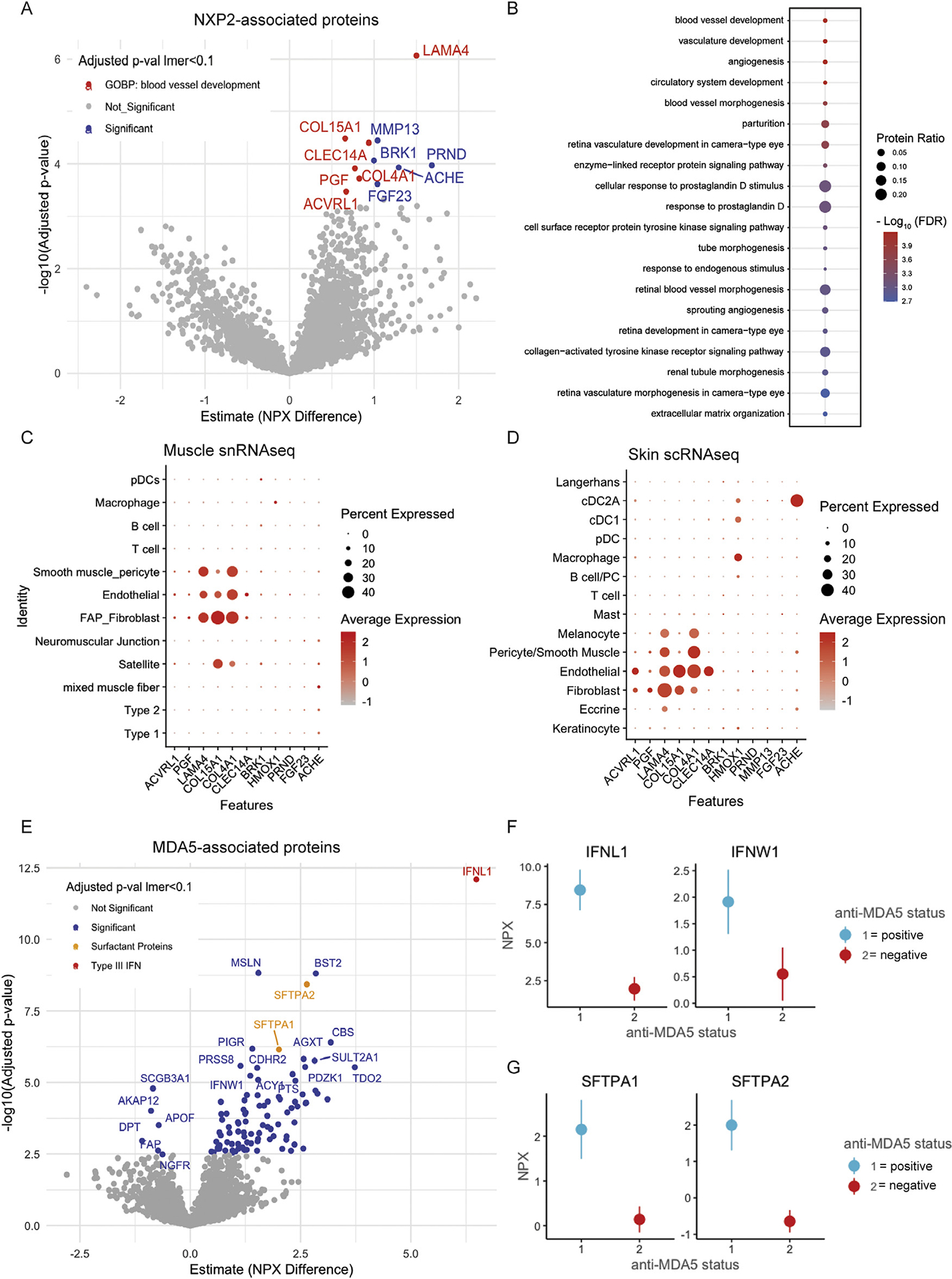
(A) Volcano plot displaying proteins differentially expressed in n = 14 patients with anti-NXP2 antibodies at baseline (BL) using a linear mixed effects (lme) model with post-hoc testing. Significance was defined as *P*-adj < .1 in the lmer and *P*-adj < .05 in post-hoc lme. Colour represents significance and red indicates proteins falling within the GOBP (Gene Ontology Biological Process) term ‘blood vessel development’. (B) Dotplot displaying pathway enrichment analysis terms associated with NXP2-associated proteins using GOBP references where dot size represents the percentage of DEPs within that term and colour represents significance defined by the FDR (false discovery rate). (C, D) Dotplots displaying normalised gene expression (indicated by colour) and per cent of cells (indicated by size of dot) expressing the genes of NXP2-associated proteins from (C) single nuclei RNA sequencing from muscle (n = 7 JDM, n = 6 CTL, 26,638 total nuclei) and from (D) single-cell RNA sequencing from skin (n = 12 JDM, n = 4 control, 91,216 total cells). (E) Volcano plot displaying proteins differentially expressed in n = 9 patients with MDA5 antibodies at BL using an lme model with post-hoc testing. Significance was defined as *P*-adj < .1 in the lme and *P*-adj < .05 in post-hoc lme. Colour represents significance and type III IFN proteins are in red and surfactant proteins in yellow for emphasis. (F) Point range plots of IFN proteins where the dot represents the fixed effect NPX estimate and the line the 95% CI summarised the NPX difference between JDM BL patients positive for MDA5 antibodies and those negative for MDA5 antibodies. (G) Same plots as E but for surfactant proteins. DEP, differentially expressed proteins; IFN, interferon; JDM, juvenile dermatomyositis; MDA5, melanoma differentiation-associated protein 5; NPX, normalised protein expression; NXP2, nuclear matrix protein 2.

To determine proteomic signatures associated with MSA subtype, we fit a model to proteins from BL JDM samples for 3 MSA groups (n = 14 anti-NXP2, n = 23 anti-TIF1*γ*, n = 9 antiMDA5). Clinical data stratified by MSA subtype are presented in Supplementary Tables S7–S9, which revealed higher skin disease activity and fewer constitutional symptoms in the TIF1y+ group, higher muscle disease and less skin disease activity in the NXP2+ group, and higher transaminase elevation and skin ulceration in the MDA5+ group. There were 11 proteins associated with anti-NXP2, no proteins associated with anti-TIF1*γ*, and 99 proteins associated with anti-MDA5 (*P*-adj < .1 in lmer, *P*-adj < .05 in post-hoc lmer, Fig 6A,E, Supplementary Tables S10, S11). There was also a higher distribution of antiNXP2+ patients from U-M; however, PCA analyses of DEPs in each group suggested that DEPs were driven more by MSA group differences than site differences (Supplementary Figs S6–S8). Despite a small number of DEP, anti-NXP2 associated proteins were significantly enriched in processes related to ‘angiogenesis’, ‘vascular development’, and ‘extracellular matrix organisation’ (Fig 6A,B, Supplementary Table S10). These proteins corresponded to specific cell types in JDM muscle and skin single-cell gene-expression datasets related to these processes: endothelial cells, pericytes, and fibroblasts (Fig 6C, D), which is intriguing given anti-NXP2 is associated with severe muscle disease, vasculopathy, and calcinosis [32]. LAMA4, a structural component of the endothelial cell basement membrane [33] with a role in trans-endothelial migration [34], was the top protein associated with anti-NXP2 JDM.

### Anti-MDA5 JDM is associated with circulating IFNL1 and surfactant proteins

Patients with anti-MDA5 antibodies had strikingly high expression of circulating IFNL1 (Fig 6E,F), a type III IFN, whose receptor is more restricted to epithelial surfaces in the lung, gut, and skin than type I IFN receptors, which are ubiquitously expressed [35]. IFNW1, a type I IFN, was also significant. Anti-MDA5 DEP were enriched in numerous metabolic processes, including oxidative stress responses (Supplementary Table S11). To our surprise, anti-MDA5 patients also had higher surfactantrelated proteins SFTPA1 and SFTPA2 (Fig 6F), primarily expressed by lung type II pneumocytes. Only 1 anti-MDA5 patient had known lung disease at sampling identified by computed tomography imaging through routine screening.

## DISCUSSION

Using exploratory plasma proteomics in a multicentre North American JDM longitudinal cohort, we identified biological pathways persistently dysregulated at 6 months despite treatment, including multiple innate immune (IL1, IL6, TLR2), redox (SOD2), proteasomal (PSME1/2, UBE2B), cell death (BCL2, CASP4) and DNA damage repair (RAD23B, PARP1) pathways. In addition to highlighting some less traditional pathways linked to JDM pathogenesis, this study also independently validated inflammatory and IFN-related proteins previously reported in JDM, including CXCL10 [17,21] and CCL7 [23]. By integrating tissue data from the HPA and JDM organ-specific single-cell datasets, we defined circulating tissue and cell-type-specific disease activity-associated proteins, including MYL3 (muscle) and IL34 (skin). We also highlighted overrepresentation of endothelial cell-specific markers, including FLT1, in proteins associated with disease activity, emphasising the central role of vasculopathy in JDM. We further described distinctive proteomic signatures of NXP2 and MDA5 JDM subgroups, laying the groundwork for better understanding JDM disease heterogeneity.

Leveraging longitudinal analysis, we uncovered proteins and pathways with subtle but persistent dysregulation that might be obscured using other research designs. Notably, redox and proteasomal signalling emerged as top dysregulated pathways, suggesting ongoing oxidative stress and antigen processing posttreatment. These findings are consistent with other studies describing proteasomal upregulation [36], oxidative stress [37], and mitochondrial dysfunction [38,39] in JDM. We also observed shifts in IL1B, CCL5, and BCL2, which appear to rise at 6 months posttreatment, raising questions regarding the role of these proteins in inflammation vs repair. Elevated circulating CCL5, an immunoregulatory chemokine, has been described not only in autoimmune myositis but also in hereditary muscle disorders and may arise from chronic muscle damage or myokine secretion [40]. CCL5 is a chemoattractant and can promote angiogenesis, revascularisation, and muscle regeneration [41]. IL1B has been previously described in JDM with low disease activity states [14], posing hypotheses of chronic muscle damage, especially as related to mitochondrial dysfunction [42,43]. Overall, these findings highlight multiple pathways with persistent dysregulation that may represent alternate treatment targets.

This study reinforces and expands upon emerging candidate markers of JDM disease activity. While proteins related to IFN signalling and muscle structure/function are most strongly associated with disease activity, disease activity-associated DEP at 6-month FU represented diverse biological processes. This suggests that alternate proteins, or a combination of proteins, may be useful to assess lower levels of disease activity. Notably, both SOD2 and PARP1 function in response to cellular injury and oxidative stress. Although SOD2 traditionally facilitates the production of reactive oxygen species (ROS), it may also buffer ROS effects under the mitochondrial unfolded protein response [44]. Notably, low SOD levels have been associated with poor prognosis and exacerbation of interstitial lung disease (ILD) in adult anti-MDA5 DM [45]. PARP1 similarly has dual roles, both as an enhancer of ROS generation and a modulator of DNA repair. Future longitudinal studies validating these biomarkers in a range of disease activity states should be prioritised, especially considering limitations of IFN and muscle-related biomarkers that may normalise more rapidly with treatment.

We also identified candidate markers reflecting organ-specific disease activity that could be useful in distinguishing disease activity from damage and monitoring of organ-specific disease activity. We found cell-type-specific muscle markers, including MYL3 for type I fibres. We identified IL-34 as a skin marker and FLT1, LIFR, and PECAM1 as endothelial markers. Endothelial-associated markers were over-represented among disease activity-associated proteins, including FLT1 (VEGFR1). Interestingly, soluble FLT1 acts as a decoy receptor, serving to inhibit rather than promote angiogenesis like the membranebound form [46]. Thus, FLT1 elevation in plasma and its association with disease activity may reflect impaired angiogenesis and vascular repair in target tissues. These findings support a central role for vascular dysregulation in JDM pathogenesis [21,22] and emphasise the critical need for vascular markers for JDM disease monitoring.

MSAs define clinical subgroups, and we found unique proteins and pathways for specific MSAs. Within anti-NXP2 JDM, we noted enrichment for angiogenesis and extracellular matrix remodelling proteins, including LAMA4. LAMA4, a laminin subunit which promotes migration of cells to inflamed tissue and regulates angiogenesis [47], showed gene expression in endothelial cells and fibroblasts in both JDM skin and muscle. It is possible that elevated LAMA4 levels may reflect more severe vasculopathy and could serve as a candidate marker of ongoing vasculopathy in anti-NXP2 JDM. In anti-MDA5 JDM, we identified high circulating levels of IFNL1, a type III IFN, which is highly expressed in lung, gut, and skin epithelium [35], all of which are clinically affected tissues in anti-MDA5 disease. IFNL1 signals with JAK1 and TYK2, making JAK inhibitors an attractive treatment for MDA5 JDM [15,35]. Small studies have indicated that anti-MDA5 JDM may have higher type I IFN-regulated gene expression in blood and muscle [10,48]; however, type I and type III IFN activate similar gene responses, so without proteomic resolution, the IFN source or type may be unclear [35]. We also identified elevated surfactant proteins (SFTPA1, SFTPA2) in anti-MDA5 JDM, despite the lack of clinically apparent lung disease. Surfactant protein A has been reported as a serum marker of nonmyositis-associated ILD [49]. Surfactant protein D is a prognostic marker for mortality in adult MDA5-ILD [50]. Since ILD is more common in anti-MDA5 JDM, higher circulating surfactant proteins may indicate subclinical lung inflammation in anti-MDA5 JDM.

A strength of our study is the inclusion of JDM plasma samples from multiple sites including CARRA Registry and Biobank; however, we had to account for site differences and not all patients had FU samples, overall decreasing power in the models. Given cost and sample constraints, we included a limited number of controls from 2 sites, but the primary focus of our study was to compare proteomic signatures between JDM samples, and we used strict cutoffs with multiple hypothesis testing and emphasised only individual proteins with large effect sizes. We had missing data for clinical disease activity measures in several patients, including the Cutaneous Dermatomyositis Disease Area and Severity Index Score activity, Manual Muscle Testing and a Subset of Eight Muscles and Childhood Myositis Assessment Scale scores, and relied on multiple physicians to score clinical measures, which may have impacted those analyses. While we were able to utilise the HPA to define tissue/cell-specific expression and used separate JDM tissue datasets to validate expression at a single-cell level in muscle and skin, we have not yet validated candidate plasma proteins in an independent JDM cohort due to limited sample availability. There were also relatively small numbers of individuals by MSA group, and we were unable to detect proteins associated with the TIF1y group, which may be due to statistical power or potentially clinical differences of systemic disease in this cohort, as skin disease activity markers may not be as represented in plasma, highlighting areas for further study. Future studies should focus on validation of markers in an independent JDM cohort and longitudinal assessments of candidate biomarkers beyond 6 months, to further support the clinical utility of these findings. Mechanistic studies to assess the role of persistently dysregulated pathways highlighted here, as well as the MSA-associated pathways, will help establish their roles in JDM pathogenesis.

By highlighting the multifaceted JDM disease biology and heterogeneity through a proteomic lens, we lay the groundwork for the development of a multiprotein biomarker panel for JDM disease monitoring. We identify key persistently dysregulated pathways in JDM despite treatment, highlight tissue and cell-type-specific markers of disease activity, and define MSA-associated proteins in anti-NXP2 and anti-MDA5 patients with JDM that may serve to distinguish disease endotypes. These findings are a key step towards the beginnings of precision medicine in JDM.

## Supplementary Material

Supplementary figures

Supplementary Table S1

Supplementary Table S2

Supplementary Table S3

Supplementary Table S4

Supplementary Table S5

Supplementary Table S6

Supplementary Table S7

Supplementary Table S8

Supplementary Table S9

Supplementary Table S10

Supplementary Table S11

Supplementary material associated with this article can be found in the online version at doi:10.1016/j.ard.2025.07.020.

## Figures and Tables

**Table 1:** Baseline demographics and autoantibody testing of participants

Characteristic	All JDM (N = 56)^a^	Healthy control (N = 8)^a^	JDM paired (N = 24)^a^
**Age (y)**	7.0 (4.0, 11.3)	7.5 (4.5, 11)	7.5 (6.0, 11.5)
**Time from symptom onset to diagnosis (mo)**	3.1 (1.6, 5.3)		2.77 (1.56, 6.23)
**Sex assigned at birth**	**N = 56** ^b^	**N = 8** ^b^	**N = 24** ^b^
Female	39 (70%)	4 (50%)	16 (66.7%)
Male	17 (30%)	4 (50%)	8 (33.3%)
**Race and ethnicity**			
White	31 (55.4%)	5 (62.5%)	15 (62.5%)
Hispanic or Latino	9 (16.1%)	0 (0.0%)	2 (8.3%)
2+ Race	8 (14.3%)	1 (12.5%)	3 (12.5%)
Black or African American	3 (5.4%)	0 (0.0%)	1 (4.2%)
Middle eastern	2 (3.6%)	0 (0.0%)	1 (4.2%)
Other race	2 (3.6%)	0 (0.0%)	1 (4.2%)
American Indian or Alaskan Native	1 (1.8%)	0 (0.0%)	1 (4.2%)
Native Hawaiian or Other Pacific Islander	0 (0.0%)	1 (12.5%)	0 (0.0%)
Asian	0 (0.0%)	1 (12.5%)	0 (0.0%)
**Treatment naïve**	48 (85.7%)		16 (67%)
**Myositis-specific antibodies** ^c^	**All JDM (N = 56)** ^b^		**JDM paired (N = 24)** ^b^
Anti-TIF1-γ (formerly p155)	23/56 (41.0%)	–	9 (37.5%)
Anti-NXP2 (formerly MJ)	14/56 (25.0%)	–	5 (20.8%)
Anti-MDA5	9/56 (16.0%)	–	6 (25.0%)
Anti-Mi2	12/56 (21.4%)	–	6 (25.0%)
MSA negative	8/56 (14.3%)	–	4 (16.7%)
**Myositis-associated antibodies** ^c^		–	
ANA	32/49 (65.3%)	–	13 (54.6%)
Anti-Ro (SSA)	8/56 (14.3%)	–	4 (16.7%)
Anti-PMSCL	2/56 (3.6%)	–	0 (0.0%)

ANA, anti-nuclear antibody; BL, baseline; HMGCR, 3-hydroxyl-3-methyl-glutaryl-coenzyme A reductase; JDM, juvenile dermatomyositis; MDA5, melanoma differentiation-associated protein 5; MSA, myositis-specific autoantibody; NXP2, nuclear matrix protein 2; PMSCL, polymyositis scleroderma; SRP, signal recognition peptide; SSA, Sjögren’s syndrome-related antigen A; SSB, Sjögren’s syndrome-related antigen B; TIF1-*γ*, transcription intermediary factor 1-gamma. Bold font is used within the table to emphasise sections.

a Median (IQR).

b n (%).

c Anti-Jo1, SRP, HMGCR, La (SSB), and Ku are excluded because no patients were positive for these antibodies.

**Table 2:** Clinical data for patients with JDM at BL and for paired JDM samples at BL and FU

Clinical characteristics	All JDM at BL (N = 56)^a^	Paired JDM at BL (N = 24)^a^	Paired JDM at FU (N = 24)^a^
Physician global VAS	6.0 (5.0, 7.0)/0	6.0 (5.0, 7.0)/0	3.0 (1.0, 3.25)/0
Muscle VAS	4.0 (3.0, 6.25)/4	4.0 (3.0, 6.0)/3	1.0 (0.0, 3.0)/3
Skin VAS	5.0 (3.0, 6.0)/4	5.0 (3.0, 6.0)/3	2.0 (0.6, 2.4)/2
Patient/parent global VAS	5.0 (4.0, 7.0)/6	6.0 (3.75, 7.0)/0	3.0 (1.0, 3.8)/2
CHAQ	1.0 (0.375, 2.125)/8	0.875 (0.375, 2.127)/1	0.125 (0.0, 0.625)/3
CDASI Activity Score	12 (6.0, 17.5)/13	11 (6.8, 16.50)/8	2.0 (1.0, 4.0)/5
CMAS	31.5 (24.3, 45.0)/30	38.5 (22.5, 48.5)/6	47.0 (43.5, 50.5)/9
MMT8	66 (49.5, 73.8)/30	71.5 (53.5, 79.3)/14	79.5 (69.75, 80.0)/8
**Laboratory tests (U/L)**			
CK	309 (82, 949)/2	115 (51, 701)/1	47.5 (34.8, 87.8)/4
LDH	520 (349, 1086)/5	551 (350, 1201)/2	578 (267, 663)/3
Aldolase	15.9 (9.10, 33.975)/8	10.7 (9.0, 30.5)/5	6.9 (5.7, 9.5)/9
AST	75 (51.00, 132.75)/1	61.0 (33.0, 126.5)/1	38.5 (25.5, 52.5)/2
ALT	41 (20.75, 98.75)/1	31.0 (17.5, 78.5)/1	21.0 (16.0, 32.0)/1
**History**	**N = 56** ^b^	**N = 24** ^b^	**N = 24** ^b^
Elevated muscle enzymes	49 (87.5%)	20 (83.3%)	–
Proximal muscle weakness	50 (89.2%)	21 (87.5%)	–
Rash consistent with JDM	50 (89.3%)	21 (87.5%)	–
Glucocorticoids/steroids	6 (10.7%)	4 (16.7%)	–
Immune suppressants^c^	5 (8.9%)	3 (12.5%)	–
**Symptoms due to JDM**			
Fever	11 (20.0%)	5 (20.8%)	0 (0.0%)
Fatigue	41 (75.9%)	16 (66.7%)	4 (16.7%)
Weight loss (>5%)	9 (17.0%)	20 (83.3%)	0 (0.0%)
Arthritis	20 (36.4%)	10 (41.7%)	2 (8.3%)
Respiratory muscle weakness	3 (5.7%)	1 (4.2%)	1 (4.2%)
Interstitial lung disease	1 (2.0%)	0 (0.0%)	2 (8.3%)
Gottron’s papules or sign	48 (85.7%)	20 (83.3%)	12 (50.0%)
Heliotrope rash	42 (76.4%)	18 (75.0%)	4 (16.7%)
Cutaneous ulceration	5 (9.1%)	3 (12.5%)	1 (4.2%)
Periungual capillary loop changes	43 (78.2%)	16 (66.7%)	14 (58.3%)
Current calcinosis	1 (3.4%)	1 (0.0%)	0 (0.0%)
**Current medications**			
Glucocorticoids/steroids	–	–	22 (91.7%)
Oral steroid dose (mg)	–	–	16.6 (9.9, 24.4)
Methotrexate	–	–	21 (87.5%)
IVIG	–	–	14 (58.3%)
MMF/MPA	–	–	3 (12.5%)
Cyclophosphamide	–	–	2 (8.3%)
Rituximab	–	–	0 (0.0%)

ALT, alanine aminotransferase; AST, aspartate aminotransferase; BL, baseline; CDASI, Cutaneous Dermatomyositis Disease Area and Severity Index Score; CHAQ, Childhood Health Assessment Questionnaire; CK, creatine kinase; CMAS, Childhood Myositis Assessment Scale; FU, follow-up; IVIG, intravenous immunoglobulin; JDM, juvenile dermatomyositis; LDH, lactate dehydrogenase; MMF, mycophenolate mofetil; MMT8, Manual Muscle Testing and a Subset of Eight Muscles; MPA, mycophenolic acid; VAS, visual analogue scale.

Bold font is used within the table to emphasise sections.

a Median (IQR)/# missing data.

b n positive (% positive); percentage is out of those with complete data; history items only collected at BL; current medications assessed at FU.

c Methotrexate (n = 5), IVIG (n = 2), plaquenil (n = 3); note that plaquenil monotherapy (n = 1) was not considered systemic immune suppressant therapy.

## Data Availability

Data will be deposited in NCBI Gene Expression Omnibus, an open public repository, at time of publication. Code will be available on a public GitHub repository.

## WHAT IS ALREADY KNOWN ON THIS TOPIC

- There are multiple emerging inflammatory and interferonrelated disease activity biomarkers in juvenile dermatomyositis (JDM); however, there is still a limited understanding of molecular mechanisms underlying disease heterogeneity and organspecific inflammation as well as a limited repertoire of biomarkers to monitor lower levels of disease activity and vascular health in patients with JDM posttreatment.

## WHAT THIS STUDY ADDS

- This exploratory proteomics study in a diverse, multicentre North American cohort of 56 newly diagnosed patients with JDM with 24 paired 6-month follow-up samples uncovered multiple persistently dysregulated biological pathways not targeted by current treatments, including redox, innate immune, proteasomal, cell death, and DNA damage repair signalling. By leveraging JDM single-cell datasets, candidate tissue and cell-specific proteins were identified for muscle, skin and endothelial cells, with endothelial-specific markers being over-represented in the group of global disease activity-associated proteins. Through analysing patient samples by myositis-specific autoantibody status, specific proteomic signatures were identified for NXP2+ and MDA5+ JDM reflective of clinical phenotypes.

## HOW THIS STUDY MIGHT AFFECT RESEARCH, PRACTICE OR POLICY

- These findings highlight the complex, multiorgan biological pathway dysregulation contributing to JDM disease heterogeneity, tissue-specific inflammation, and persistent disease activity, laying the groundwork for alternate treatment targets and biomarkers as well as precision medicine care for patients with JDM.
