# Supplementary material for: Discovery of tissue-specific proteomic signatures in juvenile dermatomyositis highlights pathways reflecting persistent disease activity, clinical heterogeneity, and myositis-specific autoantibody subtype
Source: Ann Rheum Dis. Author manuscript; Available in PMC 2026 Feb 22. (PMC12925510; doi:10.1016/j.ard.2025.07.020)
Supplement: Supplementary figures [file NIHMS2147268-supplement-Supplementary_figures.pdf]

Supplementary Figure S1

1A

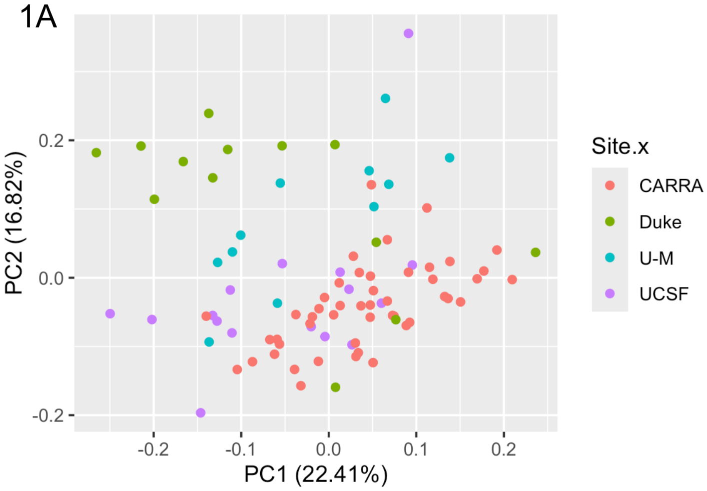

1A: PCA plot of the first 2 PC's of 88 samples where color indicates site.

1B

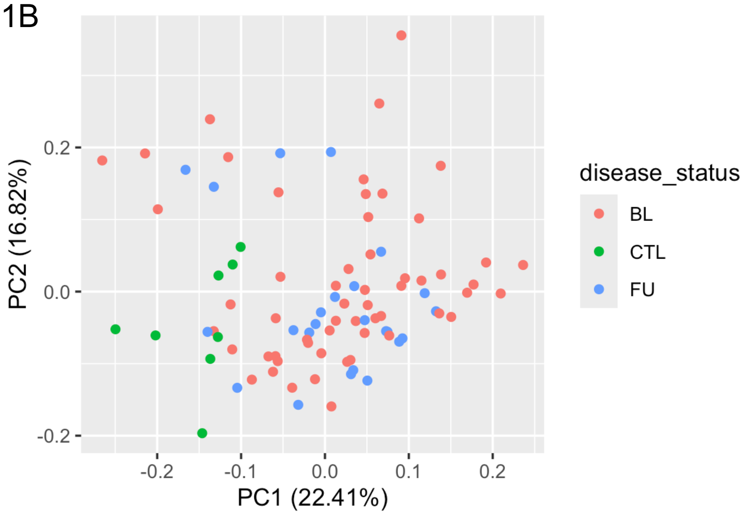

1B: PCA plot of the first 2 PC's of 88 samples where color indicates disease group (BL=baseline, FU=follow up, CTL=control).

1C

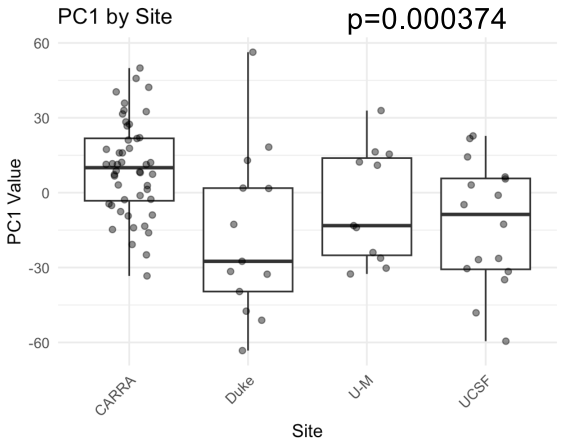

1C: Boxplot of PC1 values by site with p value of ANOVA displayed above.

1D

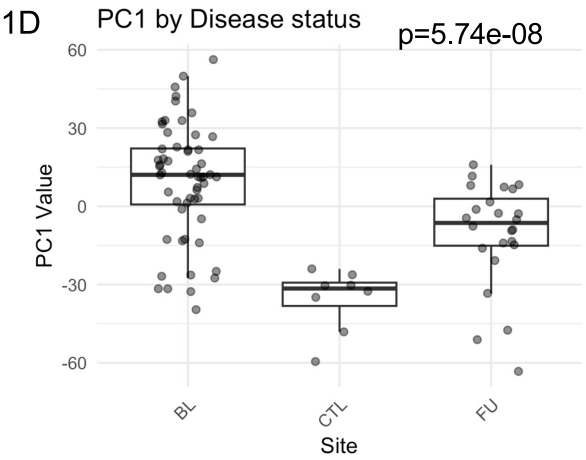

1D: Boxplot of PC1 values by disease status with p value of ANOVA displayed above.

Supplementary Figure S2

2A: All proteins in BL & CTL samples

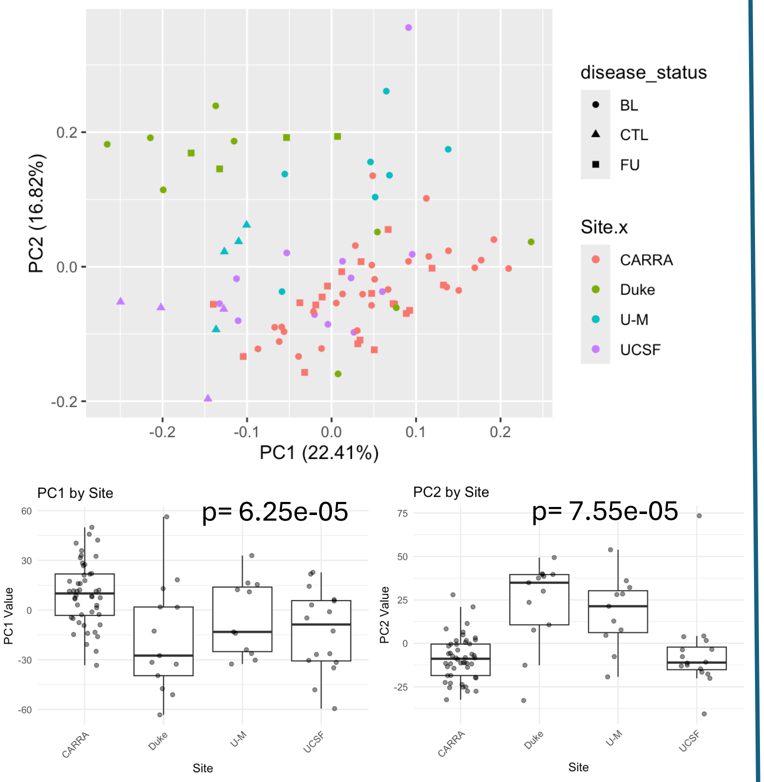

2A: PCA plot of all proteins in BL and CTL samples colored by site and boxplots of PC1 and PC2 by site with p-values of ANOVA testing displayed above.

2B: Significant proteins only in BL & CTL samples

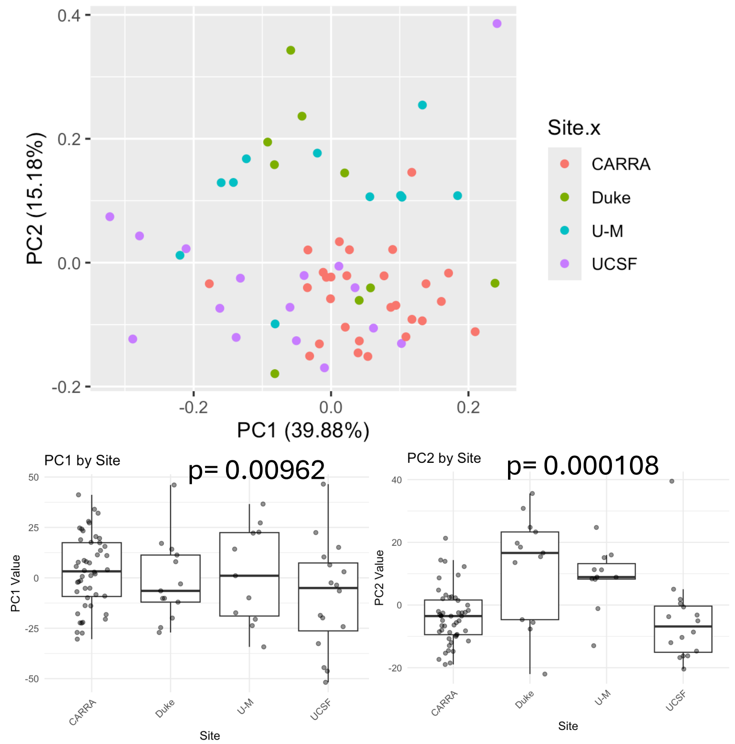

2B: PCA plot of significant proteins from BL v CTL analysis colored by site and boxplots of PC1 and PC2 by site with p-values of ANOVA testing displayed above.

2C: All proteins in BL & CTL samples

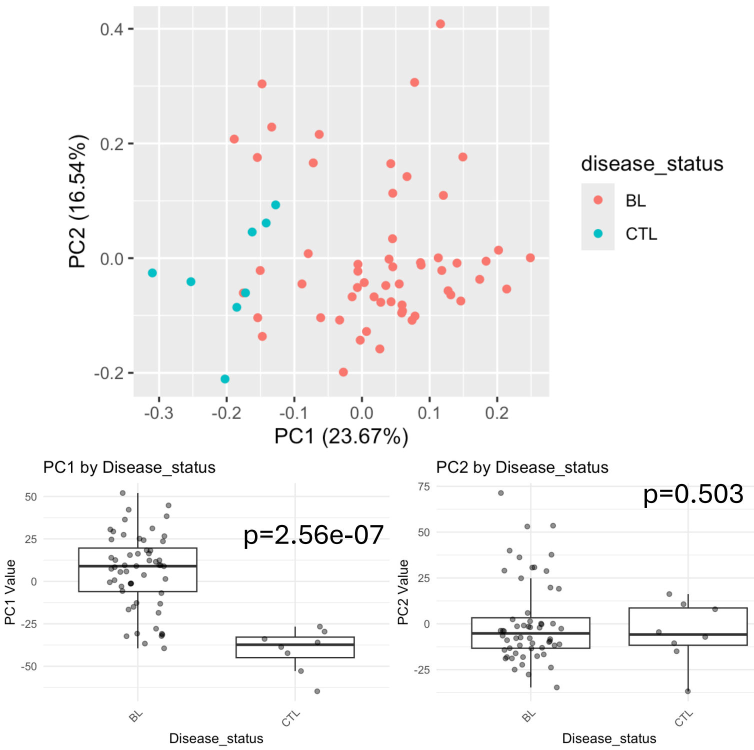

2C: PCA plot of all proteins in BL and CTL samples colored by disease status and boxplots of PC1 and PC2 by disease status with p-values of ANOVA testing displayed above.

2D: Significant proteins only in BL & CTL samples

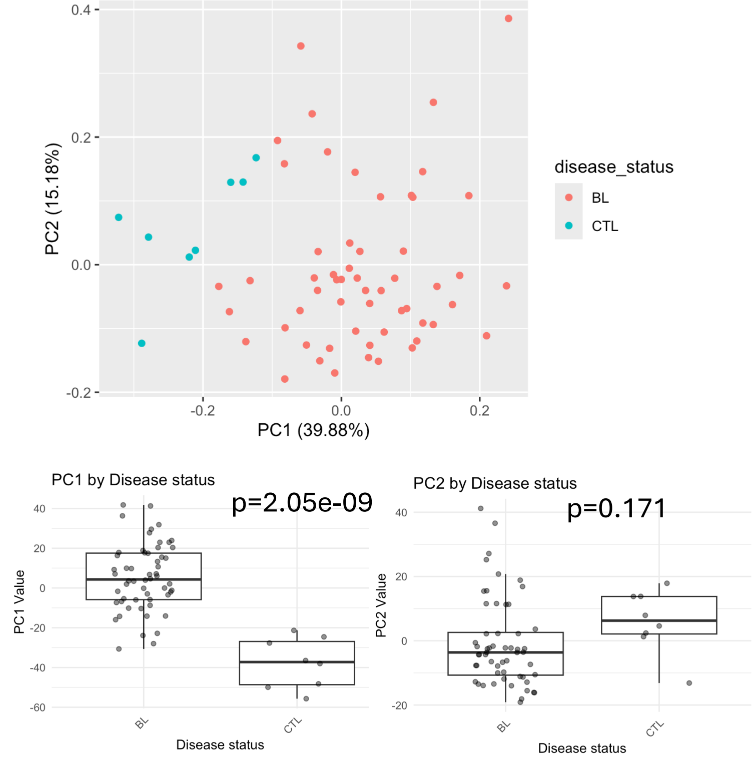

2D: PCA plot of significant proteins from BL v CTL analysis colored by disease status and boxplots of PC1 and PC2 by disease status with p-values of ANOVA testing displayed.

Supplementary Figure S3

3A: All proteins in FU & CTL samples

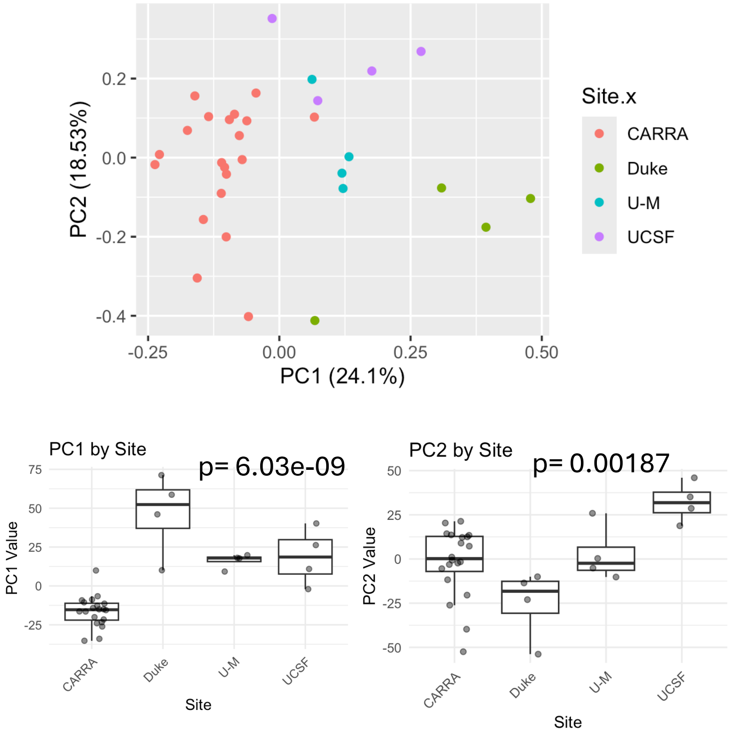

3A: PCA plot of all proteins in FU and CTL samples colored by site and boxplots of PC1 and PC2 by site with p-values of ANOVA testing displayed above.

3B: Significant proteins only in FU & CTL samples

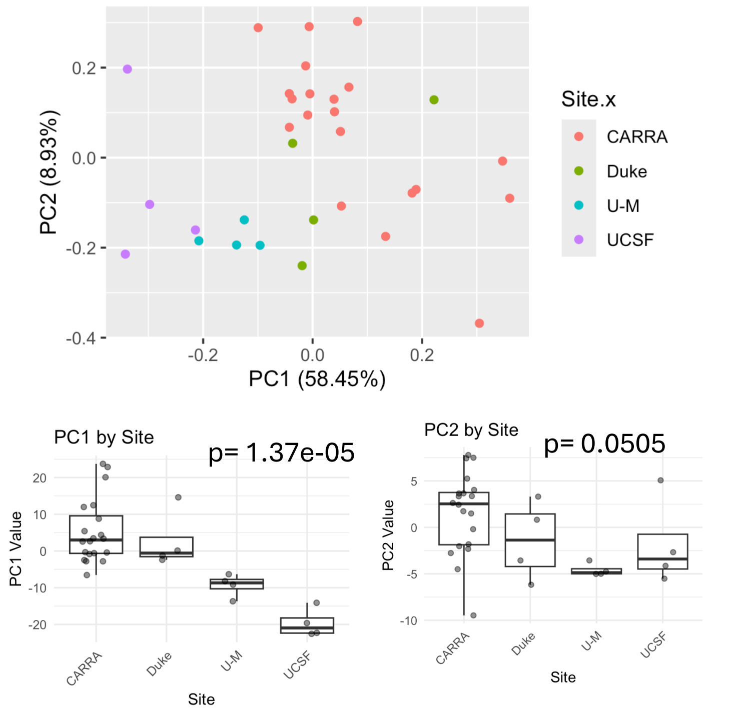

3B: PCA plot of significant proteins from FU v CTL analysis colored by site and boxplots of PC1 and PC2 by site with p-values of ANOVA testing displayed above.

3C: All proteins in FU & CTL samples

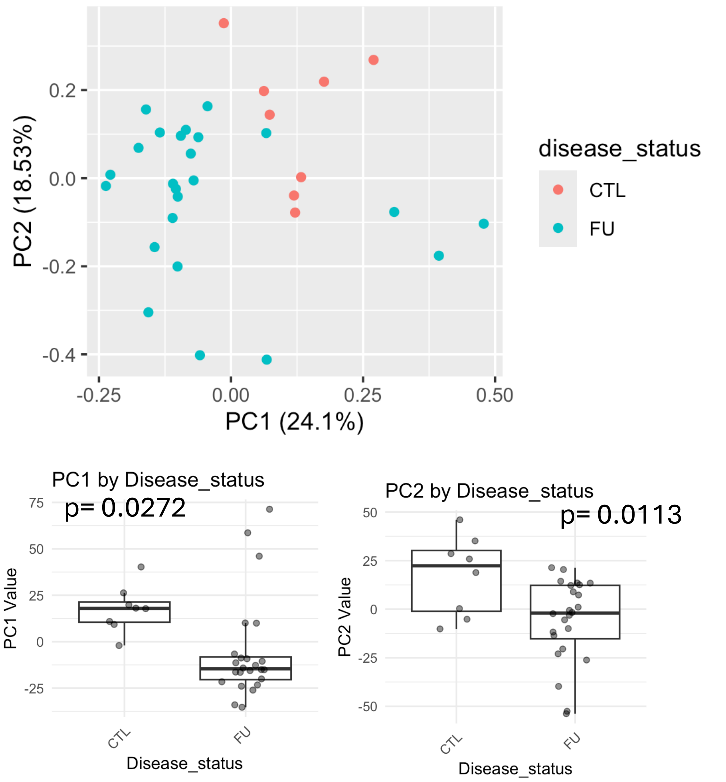

3C: PCA plot of all proteins in FU and CTL samples colored by disease status and boxplots of PC1 and PC2 by disease status with p-values of ANOVA testing displayed above.

3D: Significant proteins only in FU & CTL samples

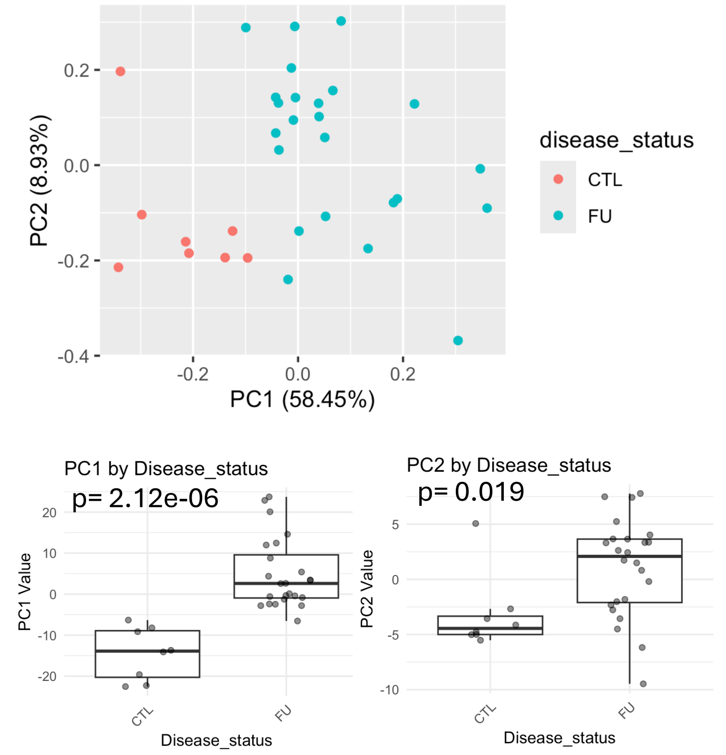

3D: PCA plot of significant proteins from FU v CTL analysis colored by disease status and boxplots of PC1 and PC2 by disease status with p-values of ANOVA testing displayed.

Supplementary Figure S4: IL1B expression in PBMC scRNAseq data

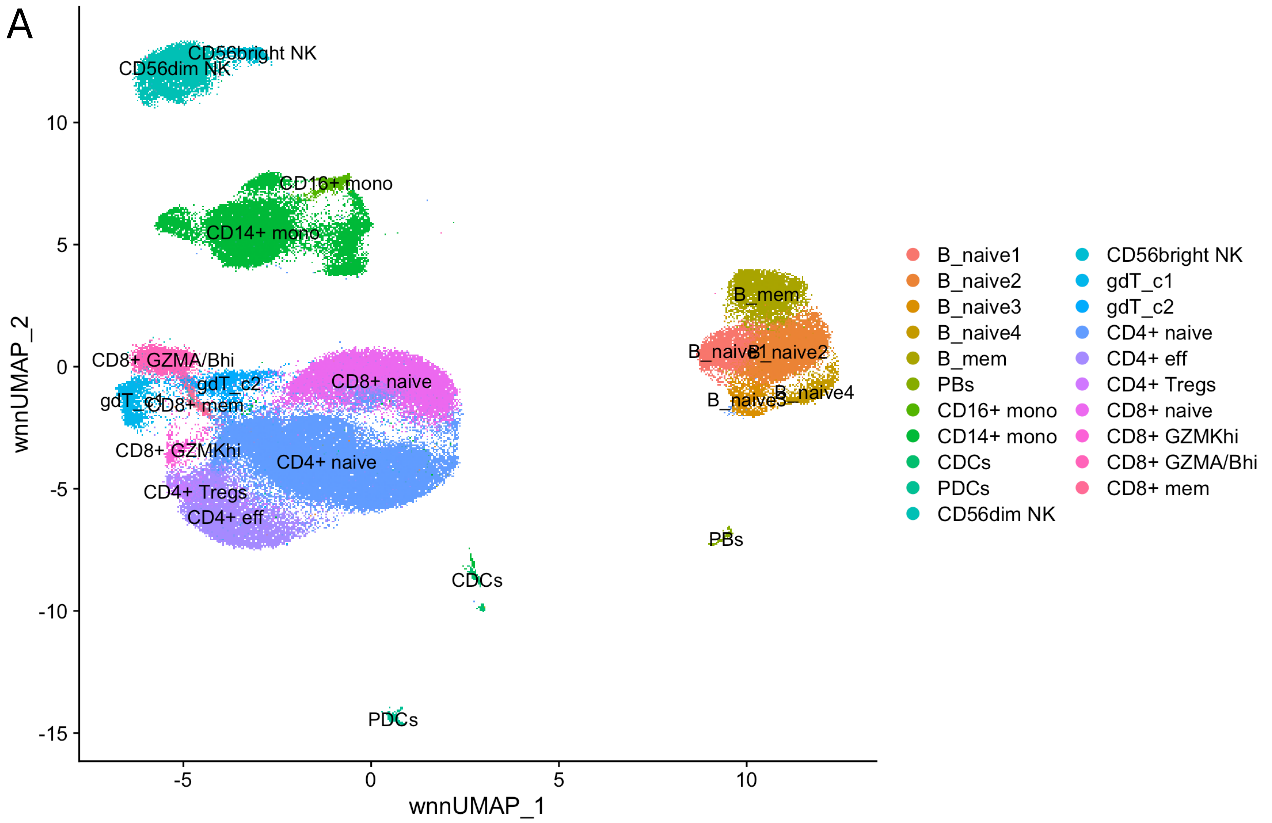

A. UMAP from Rabadam, Wibrand, et al. JCI Insight 2024 with annotated cell types.

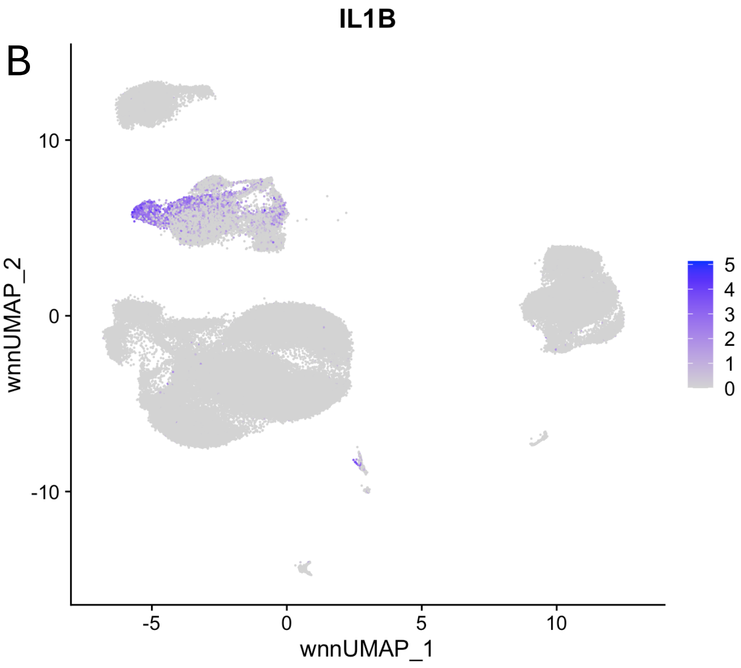

B. UMAP colored by IL1B expression showing restricted expression in CD14+ monocytes

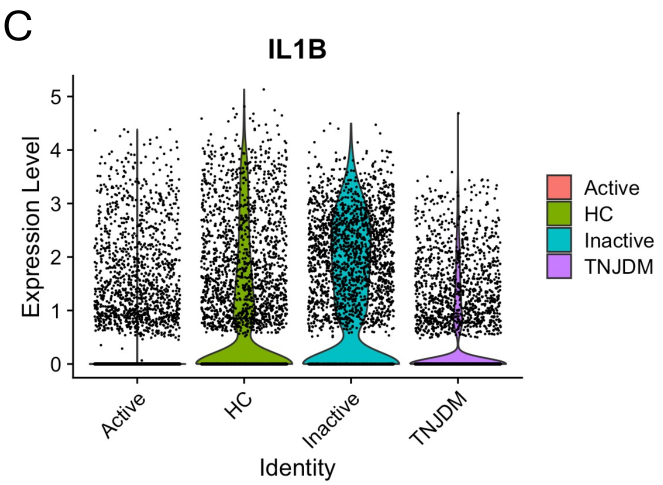

C. Violin plot of IL1B expression in CD14+ monocytes grouped by disease activity group where TNJDM = treatment-naïve JDM and inactive JDM have inactive disease off medication.

Supplementary Figure S5: Sensitivity analysis of FU v CTL samples removing 5 patients who received MMF, MPA, or CYC and comparison to original results

A

Log-Log plot showing 160 common DEPs in sensitivity analysis (x-axis) and original analysis (y-axis) demonstrations similar estimates and direction of expression in both analyses

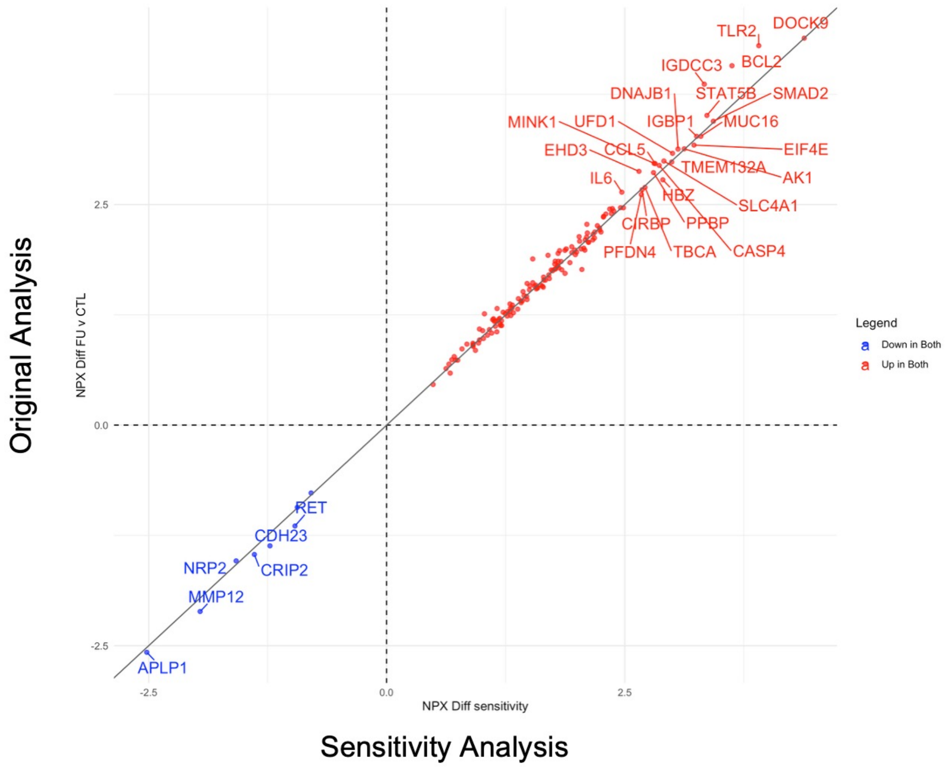

B

Estimates for sensitivity analysis with n=5 FU JDM removed that received MMF/MPA or CYC

Original estimates for these two proteins in FU v CTL analysis

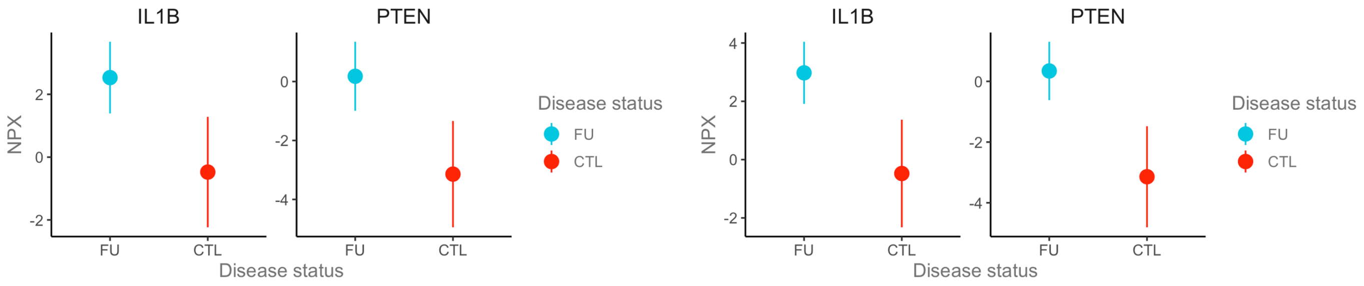

SF5: A. A linear mixed effects model using site as a covariate was performed after removing 5 patients who received MMF, MPA or cyclophosphamide. 160/232 proteins were still differentially expressed with similar estimates and the same direction of expression. **B.** IL1B and PTEN were among top proteins highlight in Fig 2E that did not meet significance, however, when plotting the estimates between disease groups this appeared to be due to loss of power by reducing total JDM cohort from N=24 patients to N=19 patients, with the estimate for IL1B decreasing but similar confidence intervals, and the confidence intervals for PTEN increasing.

Supplementary Figure S6

6A: All proteins in BL samples

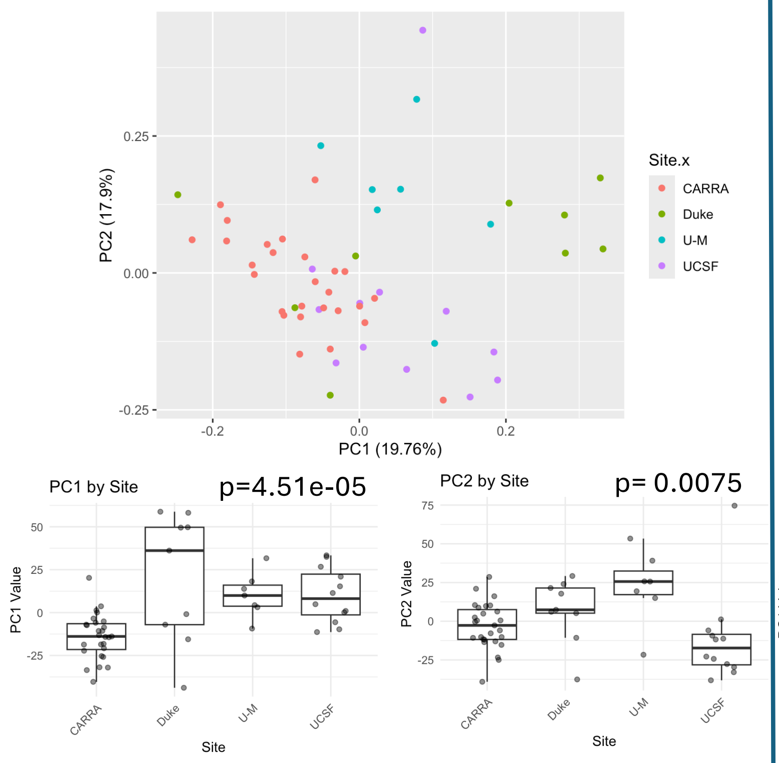

6A: PCA plot of all proteins in BL samples colored by site and boxplots of PC1 and PC2 by site with p-values of ANOVA testing displayed above.

6B: TIF1y proteins in BL samples

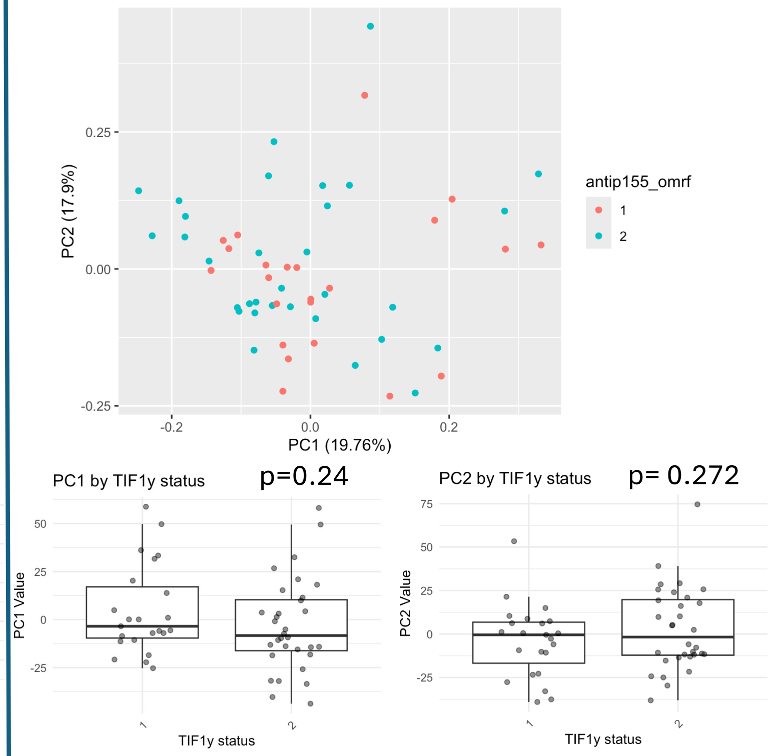

6B: PCA plot of TIF1y proteins in BL samples colored by antibody status (1=positive, 2=negative) and boxplots of PC1 and PC2 by TIF1y status with p-values of ANOVA testing displayed above.

6C: NXP2 proteins in BL samples

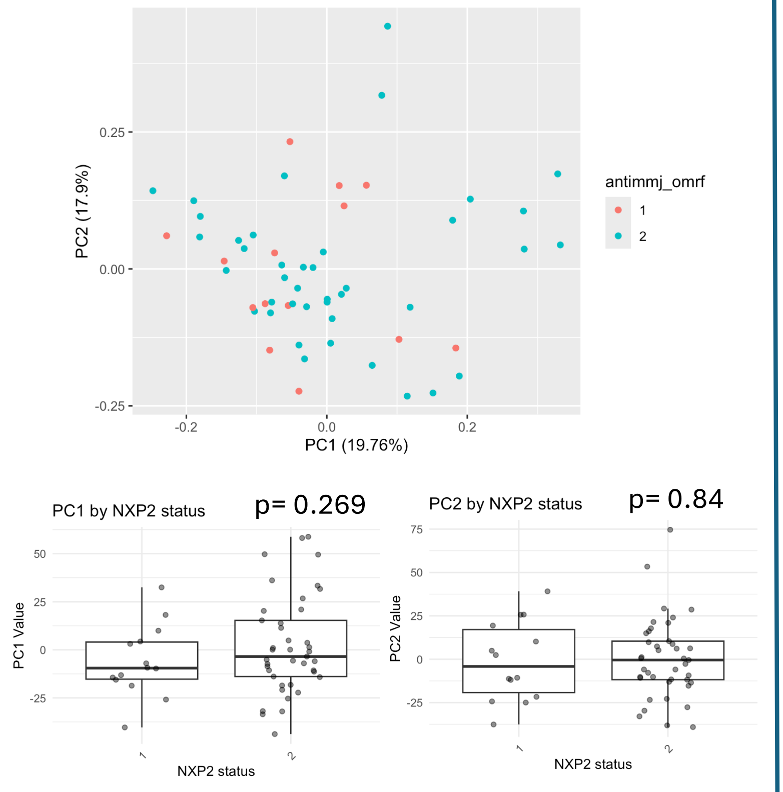

6C: PCA plot of NXP2 proteins in BL samples colored by antibody status (1=positive, 2=negative) and boxplots of PC1 and PC2 by NXP2 status with p-values of ANOVA testing displayed above.

6D: MDA5 proteins in BL samples

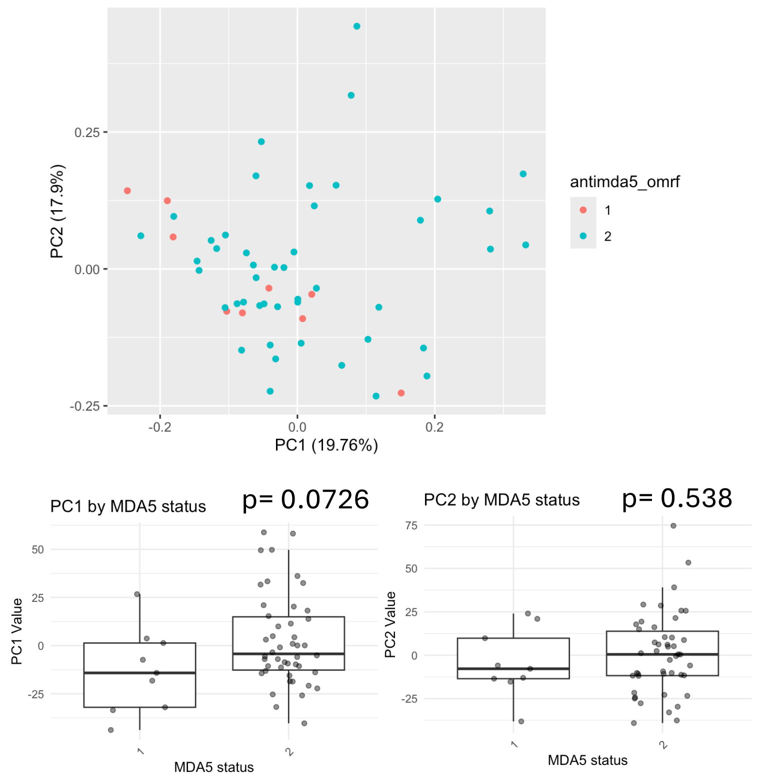

6D: PCA plot of MDA5 proteins in BL samples colored by antibody status (1=positive, 2=negative) and boxplots of PC1 and PC2 by MDA5 status with p-values of ANOVA testing displayed above.

Supplementary Figure S7: Proteins significantly associated with NXP2+ JDM(n=11 significant proteins) in BL samples evaluated by PCA by site and NXP2 status

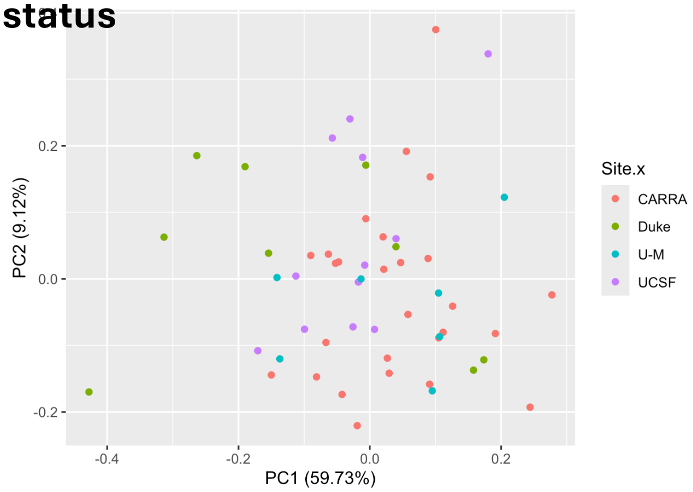

7A: PCA plot of the first 2 PC's of samples where color indicates site.

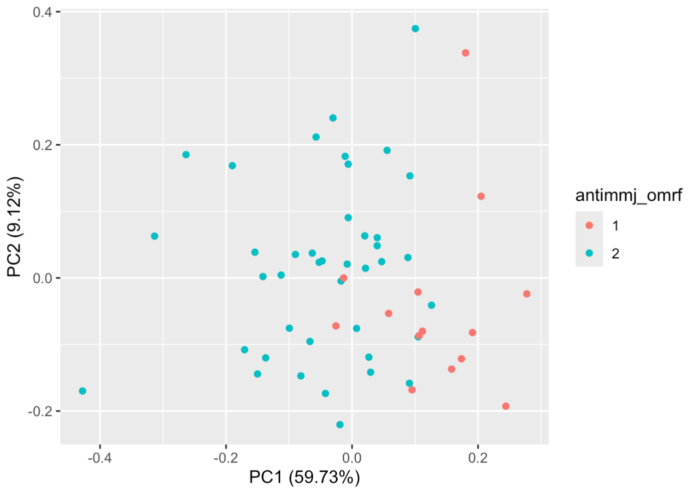

7B: PCA plot of the first 2 PC's of samples where color indicates NXP2 status (1=positive, 2=negative).

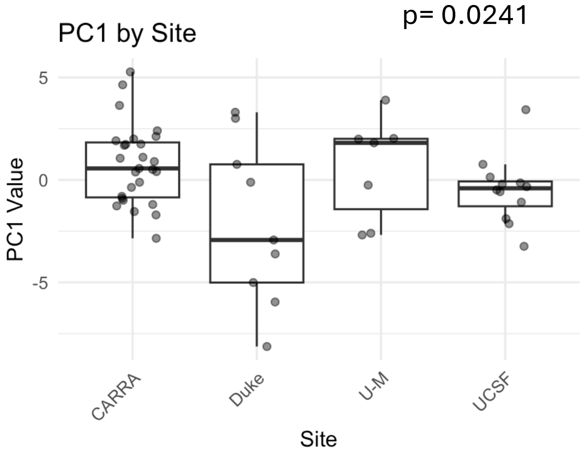

7C: Boxplot of PC1 values by site with p-value of ANOVA displayed above.

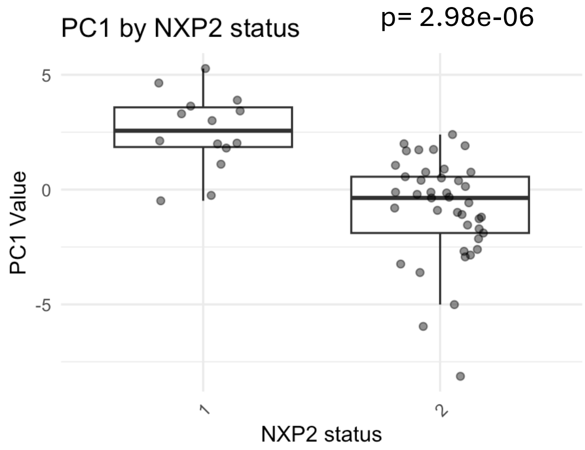

7D: Boxplot of PC1 values by NXP2 status with p-value of ANOVA displayed above.

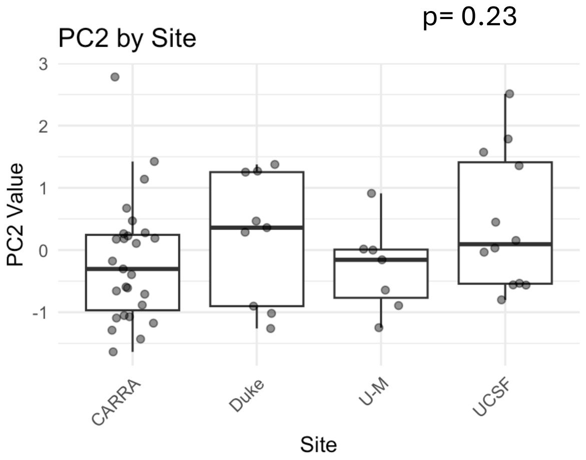

7D: Boxplot of PC2 values by site with p-value of ANOVA displayed above.

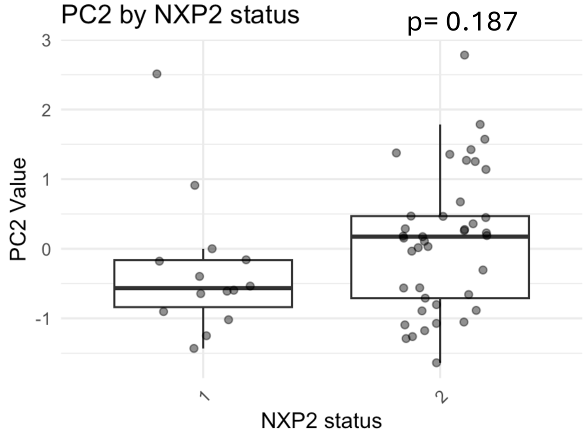

7E: Boxplot of PC2 values by NXP2 status with p-value of ANOVA displayed above.

Supplementary Figure S8: Proteins significantly associated with MDA5+ JDM (n=99 significant proteins) evaluated by PCA by site and MDA5 status

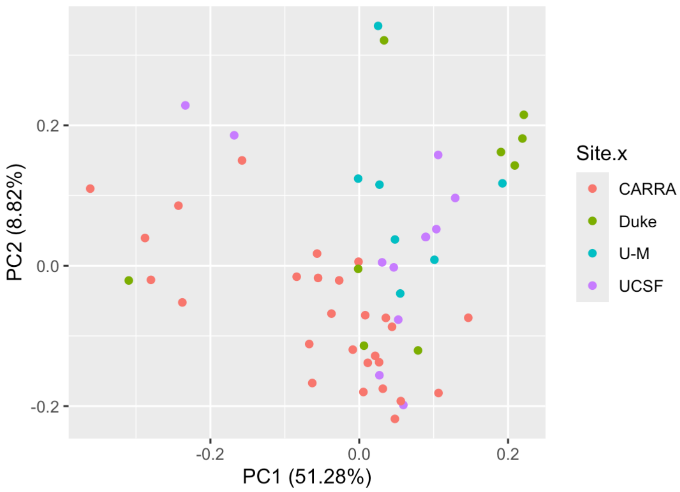

8A: PCA plot of the first 2 PC's of samples where color indicates site.

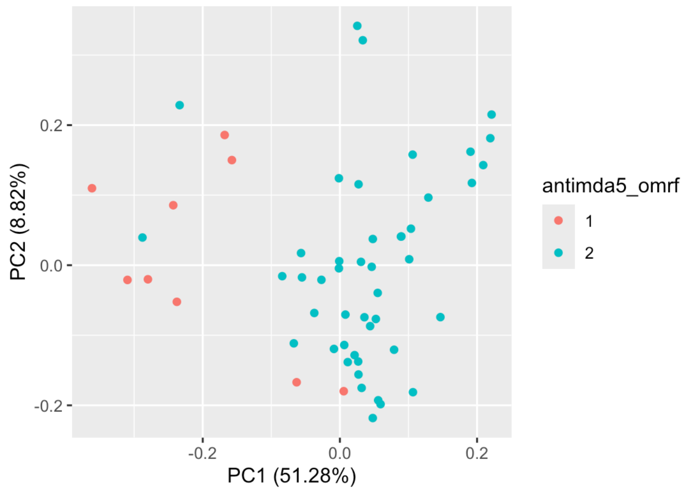

8B: PCA plot of the first 2 PC's of samples where color indicates MDA5 status (1=positive, 2=negative).

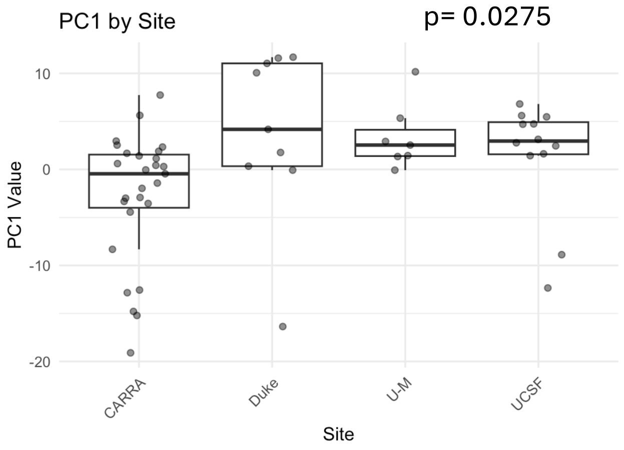

8C: Boxplot of PC1 values by site with p-value of ANOVA displayed above.

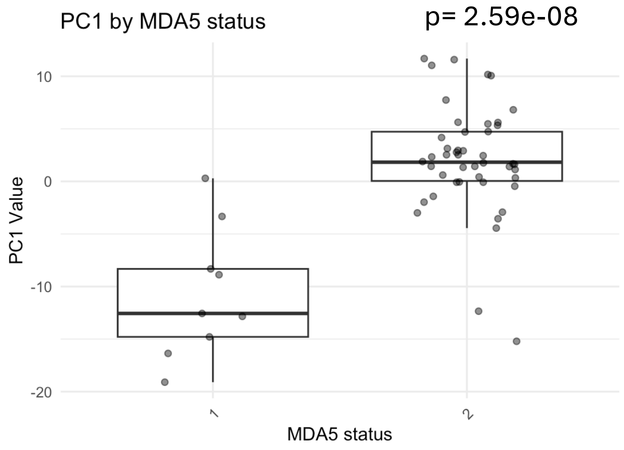

8D: Boxplot of PC1 values by MDA5 status with p-value of ANOVA displayed above.

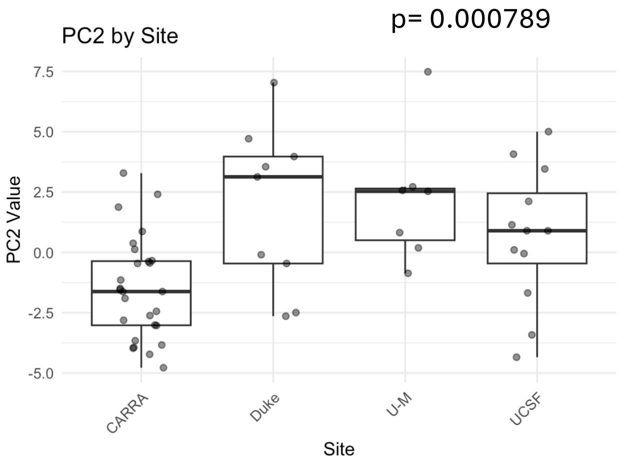

8E: Boxplot of PC2 values by site with p-value of ANOVA displayed above.

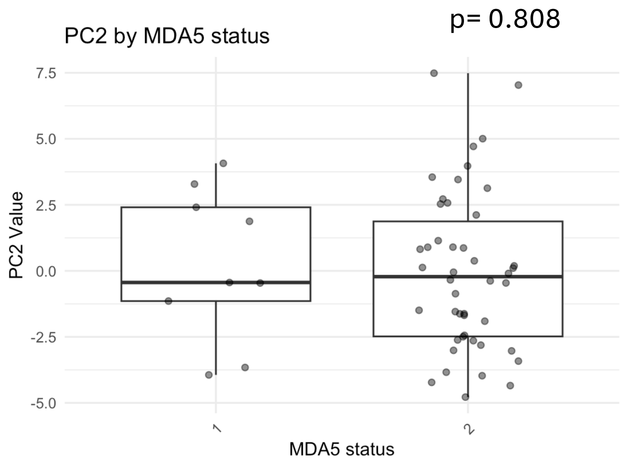

8F: Boxplot of PC2 values by MDA5 status with p -value of ANOVA displayed above.
